# Novel β-cyclodextrin–eosin conjugates

**DOI:** 10.3762/bjoc.13.52

**Published:** 2017-03-15

**Authors:** Gábor Benkovics, Damien Afonso, András Darcsi, Szabolcs Béni, Sabrina Conoci, Éva Fenyvesi, Lajos Szente, Milo Malanga, Salvatore Sortino

**Affiliations:** 1 CycloLab, Cyclodextrin R&D Ltd, Budapest, H-1097 Illatos út 7, Hungary; 2 Department of Organic Chemistry, Faculty of Science, Charles University in Prague, Hlavová 8, 128 43, Prague 2, Czech Republic; 3 Laboratory of Photochemistry, Department of Drug Sciences, University of Catania, I-95125 Viale A. Doria 6, Italy; 4 Department of Pharmacognosy, Semmelweis University, H-1085 Üllői út 26, Hungary; 5 STMicroelectronics, Stradale Primosole 50, I-95121, Catania, Italy

**Keywords:** β-cyclodextrins, fluorescence, photodynamic therapy, photosensitizers, singlet oxygen, xanthene

## Abstract

Eosin B (EoB) and eosin Y (EoY), two xanthene dye derivatives with photosensitizing ability were prepared in high purity through an improved synthetic route. The dyes were grafted to a 6-monoamino-β-cyclodextrin scaffold under mild reaction conditions through a stable amide linkage using the coupling agent 4-(4,6-dimethoxy-1,3,5-triazin-2-yl)-4-methylmorpholinium chloride. The molecular conjugates, well soluble in aqueous medium, were extensively characterized by 1D and 2D NMR spectroscopy and mass spectrometry. Preliminary spectroscopic investigations showed that the β-cyclodextrin–EoY conjugate retains both the fluorescence properties and the capability to photogenerate singlet oxygen of the unbound chromophore. In contrast, the corresponding β-cyclodextrin–EoB conjugate did not show either relevant emission or photosensitizing activity probably due to aggregation in aqueous medium, which precludes any response to light excitation.

## Introduction

Cyclodextrins (CDs) are cyclic oligosaccharides able to form host–guest inclusion complexes with drugs and this property can be utilized to protect the guest molecules from oxidation and degradation, to enhance their solubility and bioavailability, or to use the CD host as a drug carrier [1]. The potential application of these macrocyclic sugars in such pharmaceutical formulations required the understanding of the mechanism of their action and their fate inside living cells. The fluorescent labeling of CDs enables their tracking and visualization in biological media and provides useful information about their cell-membrane-penetration ability [2–3]. Besides, fluorophore-appended CDs have been extensively studied and successfully utilized in photodynamic therapy (PDT). This minimally invasive therapeutic approach has proven to be very well-suited for cancer and bacterial diseases treatment. The PDT is based on the combination of three main components: visible light, a photosensitizer (PS) and molecular oxygen [4–5]. After being excited with visible light, the PS – while reverting to the ground state – transfers the energy of its lowest excited triplet state to nearby molecular oxygen. This leads to an in situ generation of singlet oxygen (^1^O_2_), which is the main responsible species for cytotoxic reactions in cells [6]. Singlet oxygen offers important advantages over conventional drugs as it: i) potentially attacks biological substrates of different nature (i.e., lipids, proteins, and DNA), ii) does not suffer from multidrug resistance (MDR) problems, and iii) due to its short half-life (<0.1 ms) and lack of charge, it diffuses in the cellular environment over short distances (few tens of nm) resulting in negligible systemic side effects.

For PDT applications CDs have been conjugated with porphyrin [7] and protoporphyrin (5-aminolevulinic acid) [8] in order to enhance the membrane penetration of the PS (or its prodrug), to increase the aqueous solubility and to prevent the undesired aggregation of the PS inside the cell. Another advantage offered by the covalently connected CD–PS systems is the encapsulation of hydrophobic photoactivatable drug molecules in the CD cavity, enabling thereby the application of these systems in combined phototherapies. This approach has recently led to self-assembled systems based on a porphyrin–β*-*CD conjugate and a tailored nitric oxide photoreleaser forming a strong inclusion complex with the β-CD cavity [9]. This supramolecular nanoassembly simultaneously releases cytotoxic ^1^O_2_ and nitric oxide under illumination with visible light, resulting in amplified cancer-cell mortality [9]. By attaching porphyrin to γ-CD and dimeric β-CD, which are both able to form inclusion complexes with cytotoxic drug molecules such as doxorubicin and paclitaxel, nanocarriers with multimodal therapeutic effects (PDT and chemotherapy) have been developed by Král et al. [10–11]. The results achieved by these groups clearly demonstrated the numerous advantages of the PS–CD coupling. However, despite the number of porphyrinoid PSs conjugated with CDs [12–20], literature on xanthene-type PSs in conjugation with CDs is very scarce. The reasons behind this fact are most probably the difficulties in selective CD functionalization and limitations in xanthene-dye modification.

Xanthene dyes can be introduced into the CD scaffold most easily through an ester linkage between the available hydroxy groups of the CD and the carboxylic acid group of the dye [21]. These conjugates, however, cannot be used in biomedical applications because of the lability of the ester bond towards enzymatic degradation. Any cleavage of the conjugate would result in an increased free dye concentration in the studied medium and consequently it would lead to false positive results in microscopic studies or in undesired aggregation of the free PS. To circumvent this drawback and to ensure the stability of the dye-appended CD derivatives, different strategies using thioureido [3] or amide [22] connections have been developed. Fenyvesi and Jicsinszky applied thioureido chemistry to attach fluoresceinyl isothiocyanate to randomly methylated 6-monoamino-β*-*CD as a possible sensor for soluble contaminants in groundwater [23]. In the conjugate obtained, the stability of the formed thioureido moiety also made possible its biological application and since the introduction of this strategy a wide variety of xanthene-dye-appended CDs have been developed [24–25] using the same methodology. However, the major drawbacks of this approach is the extremely high price of the isothiocyanate pre-modified xanthene starting materials and the harsh reaction conditions (pyridine as a solvent, reflux, additional base) necessary for the successful coupling. These factors called for an easy-to-perform and widely applicable approach towards xanthene-dye-modified CDs. This was achieved with the aid of 4-(4,6-dimethoxy-1,3,5-triazin-2-yl)-4-methylmorpholinium chloride (DMTMMCl), a coupling reagent frequently used in peptide synthesis. Malanga et al. recently reported on the preparation of xanthene-dye-appended CDs with the most commonly used fluorescent probes rhodamine and fluorescein (Flu, **1**). The conjugation of the 6-monoamino*-*β-CD scaffold was achieved through amide-bond formation between the amino-CD and the carboxylic group of the xanthene dyes [26]. This strategy does not require the laborious isothiocyanate functionalization and it can be applied to all xanthene dyes having a carboxylic function. Encouraged by these results we decided to use this method for the coupling with xanthene dyes which upon light irradiation are able to generate cytotoxic ^1^O_2_. The coupled products would thus represent a new family of photosensitizers, the eosin-CDs (Eo–CDs). Although the photobactericidal activity of Eo dyes is well known from the literature [27–28], to the best of our knowledge Eo–CD conjugates have not been prepared up to date.

## Results and Discussion

### Synthesis of eosin Y (EoY, **2**) and eosin B (EoB, **4**)

**Figure 1:**
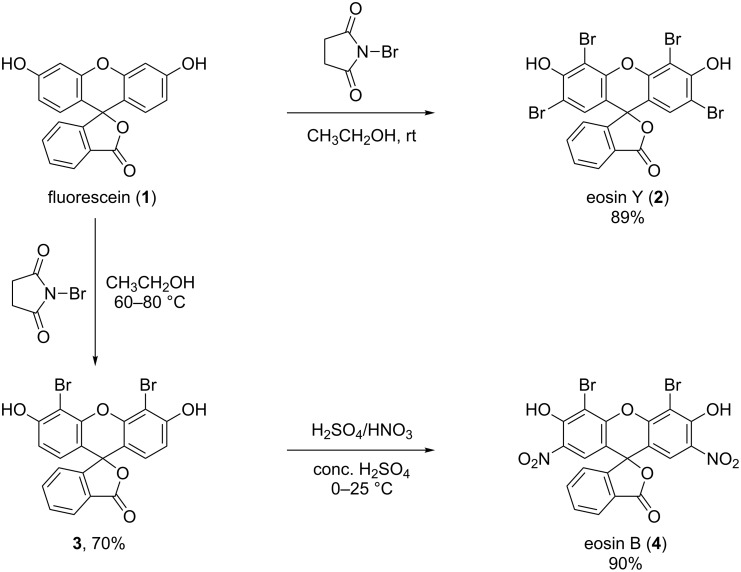
Reaction scheme for the synthesis of eosin Y (**2**) and eosin B (**4**).

The purity of the commercially available dyes eosin Y and eosin B, purchased from different providers (two different sources for each dye were tested) was not suitable for the preparation of the β-CD conjugates. The ^1^H NMR spectra and TLC analysis of the commercially available dyes are provided in Supporting Information File 1, Figures S1 and S2, Figures S8 and S9 and Figures S41 and S42, respectively. Therefore, both dyes were freshly synthesized starting from fluorescein (Flu, **1**). Although the described synthetic procedures for the preparation of eosin dyes commonly use Br_2_, herein the less hazardous *N*-bromosuccinimide (NBS) was used as the source of bromine. Thus, eosin Y (**2**) was prepared in a single step from **1** in ethanol in the presence of NBS (Figure 1). The detailed description of the syntheses is reported in Supporting Information File 1.

The synthesis of EoB (**4**) was performed in two steps: first, dibromofluorescein (**3**) was synthesized through partial bromination of **1** with NBS in acetic acid (Figure 1). However, under the selected reaction conditions, a three-component mixture of mono-, di- and tribromofluorescein was obtained and the isolation of the targeted dibrominated product **3** from this crude was extremely laborious and low yielding. On the other hand, when using NBS in a slight excess (2.5-fold molar excess with respect to **1**) only a two-component mixture of di- and tribromofluorescein was formed. The isolation of **3** from this crude was easily achieved through direct-phase silica gel column chromatography with isocratic chloroform/methanol elution. Herewith the overbrominated byproduct was permanently immobilized on the silica gel column and only the targeted dibromofluorescein (**3**) was eluted. The structure of compound **3** was elucidated with the aid of 1D and 2D NMR experiments (Supporting Information File 1, Figures S10–S14). In the following step, dibrominated fluorescein **3** was nitrated using standard nitration conditions to obtain the desired dye eosin B (**4**) (Figure 1). The NMR spectra showing the structure elucidation of the free dyes eosin Y (**2**) and eosin B (**4**) are shown in Supporting Information File 1, Figures S3–S7 and Figures S15–S19, respectively.

### Synthesis of eosin Y–β-CD and eosin B–β-CD conjugates

**Figure 2:**
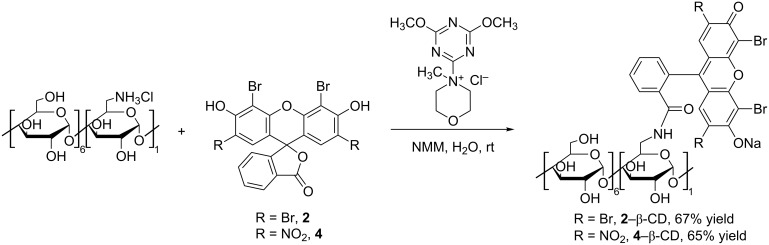
Reaction scheme for the synthesis of eosin-appended β-CDs, **2**–β-CD and **4**–β-CD (NMM: *N*-methylmorpholine).

The condensation reaction between 6-monoamino-β-CD and the synthesized dyes **2** and **4** is shown in Figure 2.

**Figure 3:**
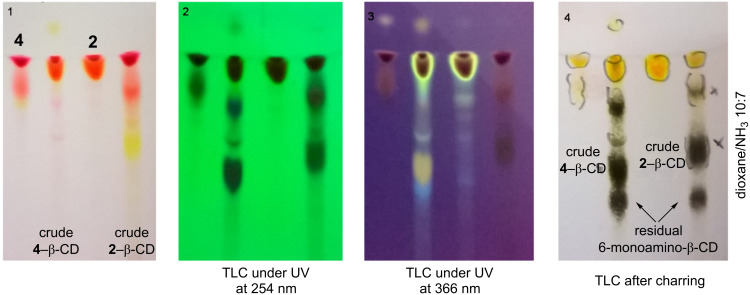
TLC analysis of the composition of the crude coupling reaction mixtures.

The reaction is promoted by the coupling agent DMTMMCl that enables an effective conjugation in water under mild reaction conditions (room temperature). Since a successful condensation requires the amino function to be in the free base form, an additional base *N-*methylmorpholine (NMM) was added. All reactants are well soluble in water resulting in a homogenous reaction mixture and in both cases the coupling proceeded at room temperature within 3 h for eosin Y (**2**) and 12 h in case of eosin B (**4**). TLC analysis of the crude reaction mixture gave the first unambiguous evidence for the successful conjugate formation as the formed products have an *R*_f_ value distinct from those of the other starting materials. Additionally the products show intensive absorbance after excitation at 254 nm and 366 nm and they are carbonizable. Thus the products exhibit the expected characteristics of a fluorescent CD conjugate (Figure 3). Work-up of the reactions included the concentration of the reaction mixtures and selective precipitation of the CD-related compounds (conjugate and unreacted starting material) with acetone. As the free dyes, NMM, DMTMM-related products and other reaction impurities are all well soluble in a water/acetone mixture, they can easily be removed by washing the precipitates with sufficient amounts of these solvents. In order to separate the target conjugates from unreacted 6-monoamino-β-CD (5–10% based on TLC evaluation, see Figure 3), the precipitate was subjected to direct-phase column chromatography using an acetonitrile (ACN)/H_2_O/NH_3_ elution mixture, in which the faster eluting component is the conjugate and the slower is the unreacted aminocyclodextrin which can be eventually recovered.

### NMR characterization of eosin-appended β-CDs, **2**–β-CD and **4**–β-CD

**Figure 4:**
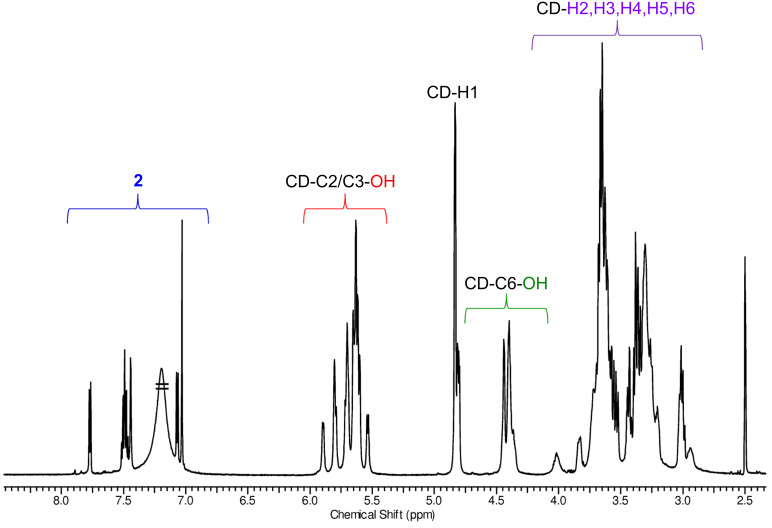
^1^H NMR spectrum of **2**–β-CD with partial assignment (DMSO-*d*_6_, 600 MHz, 298 K).

Solutions in deuterated DMSO (DMSO-*d*_6_) were used for NMR measurements, in order to preserve a molecularly dispersed form of the CD derivatives during characterization. The ^1^H NMR spectra of both conjugates have the typical fingerprint signals for the asymmetric, 6-monosubstituted β-cyclodextrins. Generally two sets of signals can be identified (Figure 4 and Figure S20 in Supporting Information File 1): the first set comprises the aromatic protons of the eosin dyes that are observed between 6.35–7.75 ppm, while the second one is formed by the CD resonances in the range of 3.0–5.9 ppm. The CD region also includes the proton resonances from the primary OH groups (4.34–4.49 ppm) and the resonances of the protons from the secondary OH groups (5.53–5.93 ppm) of the CD. DEPT-edited HSQC spectra for both products confirm the characteristic signal pattern of a 6-substituted β-CD, having the C6 carbon atom of the substituted glucose unit shifted towards lower fields (42.27 ppm) (Figure S24 and Figure S32 in Supporting Information File 1). This chemical shift of the C6 carbon atom of the substituted glucose unit is also a good indicator for the successful amide-bond formation, as this signal in the starting material, 6-monoamino-β-CD, is found at slightly higher fields – around 40 ppm. The coupling effectivity is further confirmed by the presence of a single signal at around 168 ppm in the ^13^C NMR spectra which is assigned to the carbon atom of the amide group of the conjugates (see Figures S21 and S29 in Supporting Information File 1).

In the ^1^H NMR spectra of both conjugates, the signals of the anomeric protons are well separated from the aromatic protons and from other CD-related resonances (Figure 4 and for the spectrum of conjugate **4**–β-CD, see Figure S20 in Supporting Information File 1). Therefore these protons provide a good reference for the determination of the degree of substitution (DS) of the molecules. The comparison of the intensities of the anomeric protons with those of the aromatic protons unambiguously confirms the monosubstitution pattern (DS = 1) in both cases and was further proven by electrospray ionization mass spectrometry (ESIMS) analysis of the compounds (see Supporting Information File 1). The signals in the aromatic region are well resolved (see Figures S25 and S33, Supporting Information File 1) and the in-depth analysis of the DEPT-edited HSQC spectra supported by COSY data (Figures S23 and S31, Supporting Information File 1), allowed the complete assignment of the aromatic resonances. Additional 2D NMR spectra (HMBC and ROESY) for the eosin–β-CD conjugates are also included in Supporting Information File 1, Figures S26 and S27 and Figures S34–S36).

To summarize, the compounds were prepared in good purity and a thorough investigation by NMR spectroscopy revealed that the compounds are monosubstituted on the primary side.

### Aggregation properties of eosin–β-CD conjugates by dynamic light scattering

**Figure 5:**
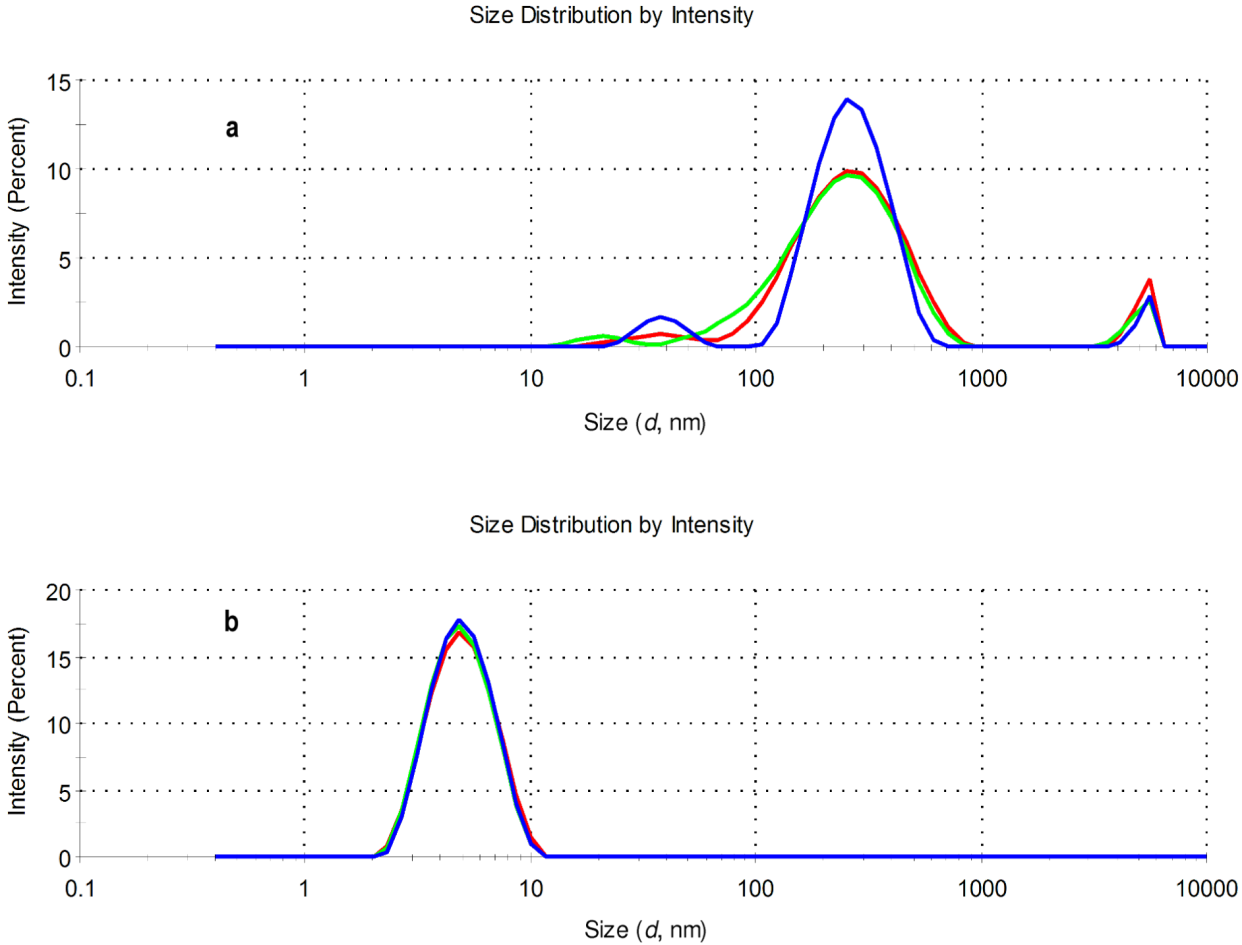
Size distributions of 1 mM aqueous solutions of conjugates **4**–β-CD (**a**) and **2**–β-CD (**b**) at 25.0 °C (pH 7) by intensity (three parallel measurements: blue, green and red lines).

For the investigation of the aggregation properties, solutions of the eosin–β-CD conjugates in water were used. A concentration of 1 mM of the conjugates Eo-β-CDs was applied to obtain reliably high scattered intensities for the characterization. Figure 5 shows that the aggregation behavior of eosin B (**4**)– and eosin Y (**2**)–β-CD conjugates significantly differ from each other. The size distribution of **2**–β-CD shows that the sample is essentially monodisperse with a peak at around 5 nm and this behavior is also observed by transforming the results into volume-related data (see Figure S39 in Supporting Information File 1). On the other hand, the conjugate **4**–β-CD is more likely to form large aggregates. Aggregate populations with sizes of approximately 100–300 nm as well as 5000–6000 nm were found for cyclodextrin conjugate with dye **4** both in the intensity vs size-distribution plot as well as in the volume plot (see Figure S39 in Supporting Information File 1).

The aggregation properties of **4**–β-CD were further confirmed by the results obtained by nanoparticles tracking analysis (Figure S40, Supporting Information File 1). The sample, under the selected experimental conditions, is rather polydisperse and differently sized populations can be detected. The strong aggregation character of **4**–β-CD can remarkably influence the spectroscopic and photophysical properties of the conjugate.

### UV–vis spectroscopic and photophysical properties of eosin–β-CD conjugates

**Figure 6:**
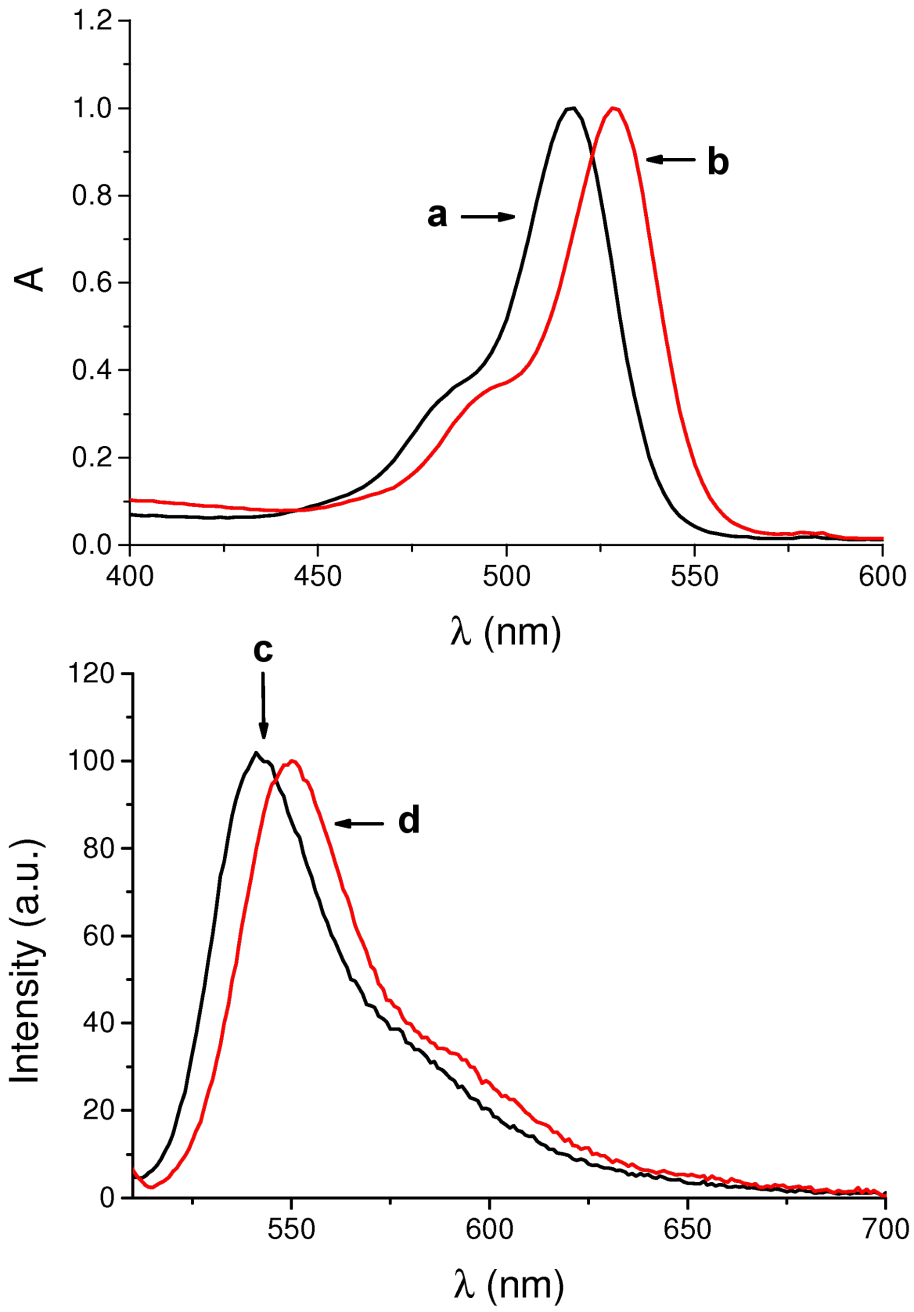
Normalized absorption spectra of aqueous solutions of (a) eosin Y (**2**) and (b) conjugate **2**–β-CD and fluorescence emission spectra of aqueous solutions of (c) **2** and (d) **2**–β-CD. λ_exc_ = 490 nm. The fluorescence spectra were recorded with the two samples having the same absorbance at the excitation wavelength.

Preliminary spectroscopic investigations on the conjugates were carried out in aqueous solutions. Figure 6 shows the absorption and fluorescence emission spectra of eosin Y conjugate **2**–β-CD and, for comparison, of the free dye **2**. Apart from a slight red shift of the absorption maximum, the absorption spectral profile in the visible region of the conjugate is similar to that of the free dye, ruling out any relevant aggregation phenomena. This hypothesis was well confirmed by the fluorescence emission spectrum, which exhibits an intense band maximum at 550 nm. The fluorescence quantum yield was Φ_f_ = 0.20, which is very close to the value reported for free eosin Y (**2**) [29].

**Figure 7:**
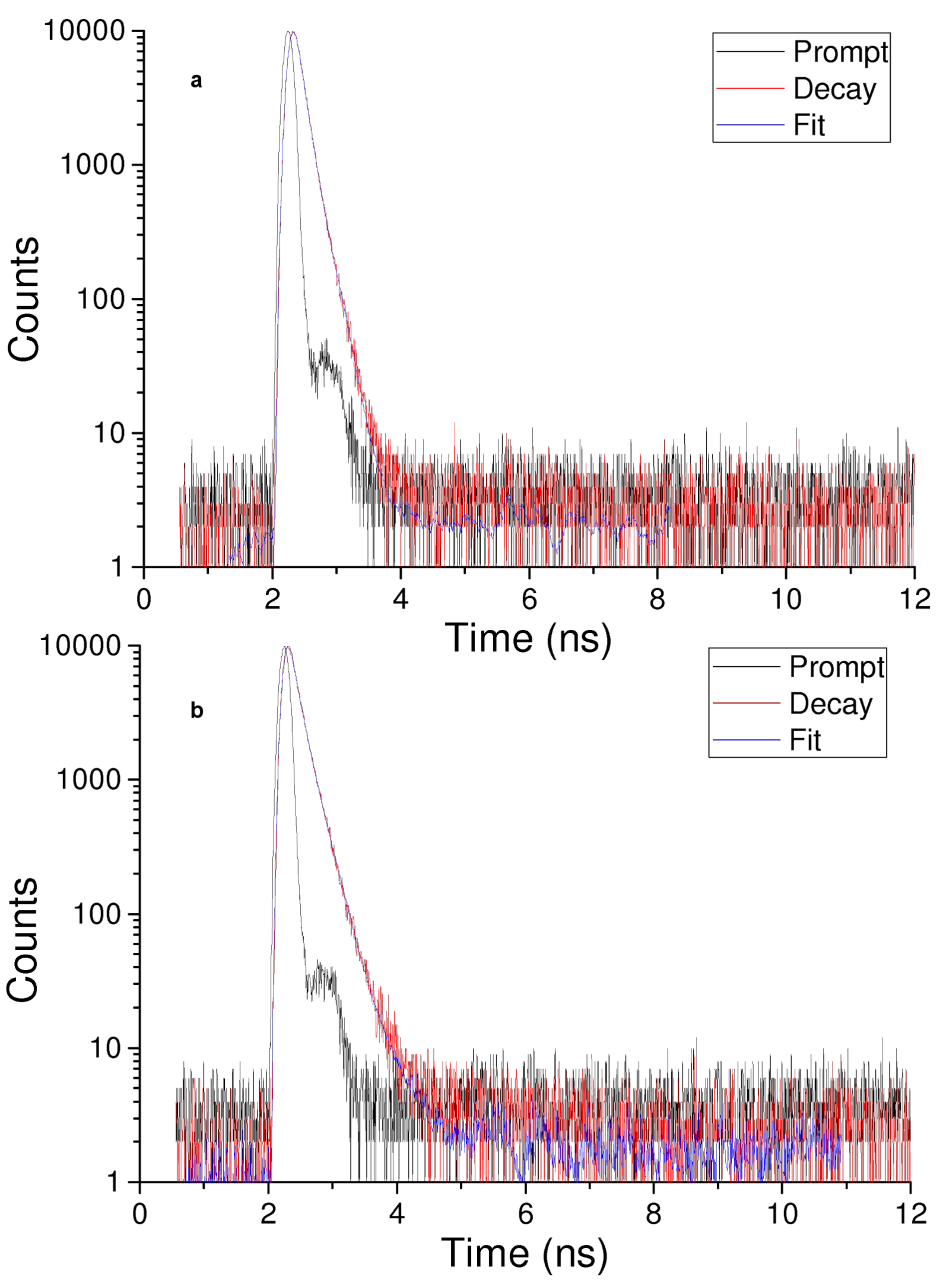
Time-resolved fluorescence observed for aqueous solutions of (a) eosin Y (**2**) and (b) the **2**–β-CD conjugate. λ_exc_ = 455 nm; λ_em_ = 570 nm.

On the other hand, the fluorescence decay of the conjugate was different from that of the isolated dye **2**. Figure 7 illustrates the fluorescence decay traces observed in both cases. The analysis of the fluorescence decay in case of **2** was fitted by a biexponential kinetic with a longer, dominant lifetime (τ) of 1.44 ns (83%) and a minor shorter component of 0.48 ns (17%). The decay of the **2**–β-CD conjugate was more complex and was fitted by a triexponential fit with lifetimes of 4.26 ns (3%), 1.77 ns (45%) and below 0.2 ns (52%). This behavior may tentatively be attributed to populations of fluorophores probably interacting in a different way with the CD cavity.

**Figure 8:**
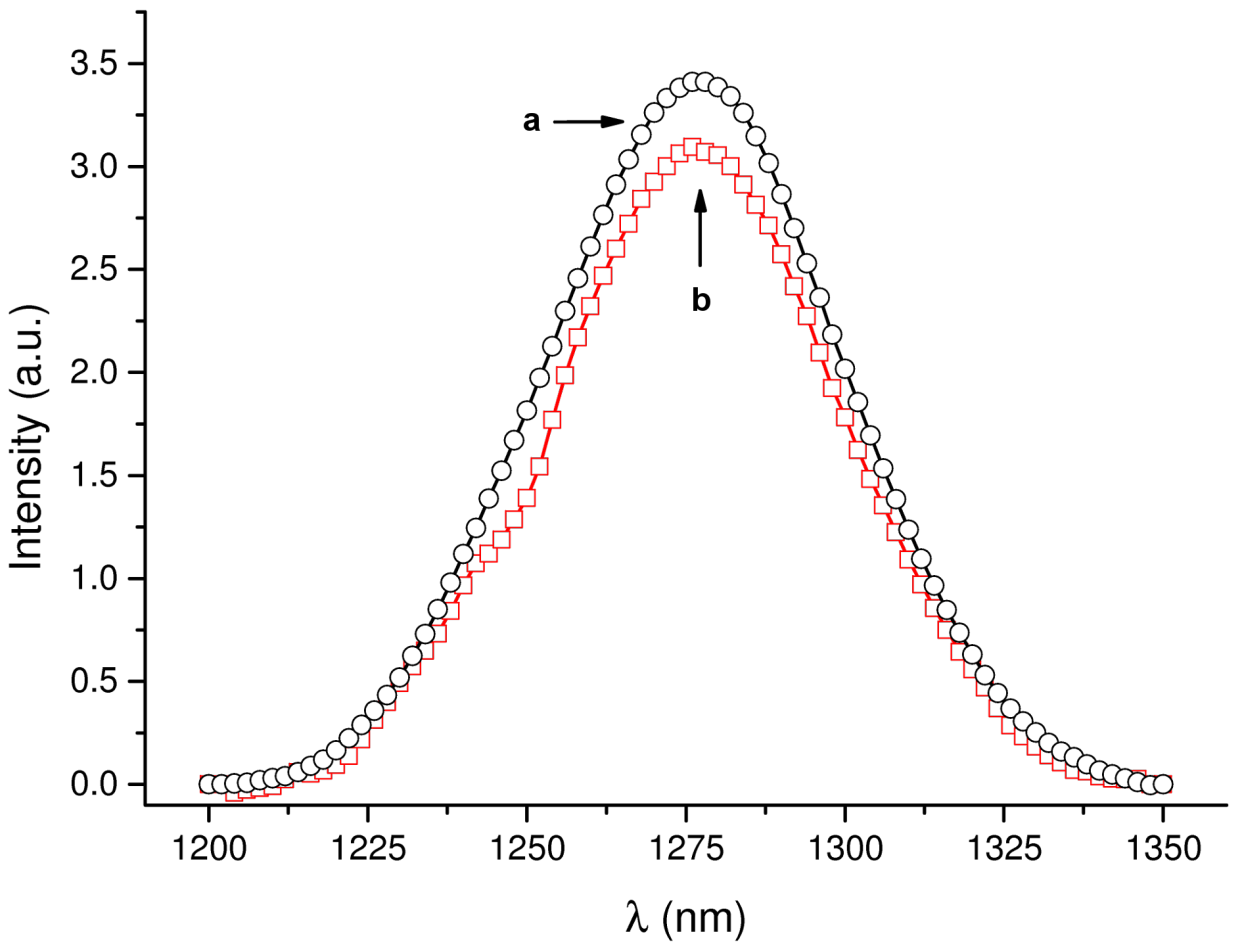
^1^O_2_ luminescence detected upon 528 nm light excitation of D_2_O solutions of (a) eosin Y (**2**) and (b) **2**–β-CD conjugate having the same absorbance at the excitation wavelength.

As outlined in the introduction, singlet oxygen, ^1^O_2_, is the key species involved in PDT and it is generated by energy transfer from the excited triplet state of a PS and the nearby molecular oxygen. Although several indirect methodologies based on suitable chemical traps are well known to detect ^1^O_2_ formation, the best experimental method to prove and quantify its production is its direct detection. It is based on the monitoring of the typical phosphorescence of ^1^O_2_ in the near-IR spectral window [30]. As shown in Figure 8, excitation of the conjugate **2**–β-CD with visible green light generates the characteristic luminescence signals with a maximum at ca. 1270 nm analogously to what observed for free dye **2**. We obtained a ^1^O_2_ quantum yield Φ_Δ_ = 0.47, that is very similar to that of the free dye in the same solvent (Φ_Δ_ = 0.49) [31]. This result excludes any significant intra- or interencapsulation of the excited triplet state of the dye within the β-CD. If this was the case, the reduced quenching by oxygen due to steric hindrance would have resulted in a much smaller value for Φ_Δ_.

The corresponding **4**–β-CD conjugate did not show either detectable fluorescence emission or ^1^O_2_ photogeneration. This is not surprising in light of the observed massive aggregation of this derivative in aqueous medium (see Figure 5). Studies currently in progress are addressed to better clarify this point and to design strategies to circumvent this drawback and results will be reported in the due course.

## Conclusion

Two novel eosin–β*-*CD conjugates have been prepared through a DMTMMCl-promoted condensation under mild reaction conditions in water. The synthesis started from the xanthene dyes that were prepared in high purity through an improved synthetic route. The prepared CD conjugates have been thoroughly characterized by 1D and 2D NMR experiments. While the eosin B (**4**)-β*-*CD conjugate was not light responsive, probably due to self-aggregation phenomena, the eosin Y conjugate **2**–β*-*CD showed excellent preservation of the photophysical properties of the dye. In fact, this molecular hybrid exhibits satisfactory fluorescence and ^1^O_2_ photogeneration quantum yields making it a suitable candidate for biomedical research studies in the field of imaging and PDT applications. The possibility to exploit the CD cavity as potential carrier site may also open intriguing prospect in multimodal therapy applications.

## Supporting Information

File 1 Syntheses, NMR spectroscopic data, ESIMS spectra, DLS study and semi-quantitative TLC of eosin-appended β-CDs.
