# Supplementary material for: Novel β-cyclodextrin–eosin conjugates
Source: Beilstein J Org Chem. 2017 Mar 15;13:543–51. doi: 10.3762/bjoc.13.52 (PMC5372748; doi:10.3762/bjoc.13.52)
Supplement: File 1 — Syntheses, NMR spectroscopic data, ESIMS spectra, DLS study and semi-quantitative TLC of eosin-appended β-CDs. [file Beilstein_J_Org_Chem-13-543-s001.pdf]

Supporting Information
for
Novel β-cyclodextrin–eosin conjugates

Gábor Benkovics[1,2], Damien Afonso[3], András Darcsi[4], Szabolcs Béni[4], Sabrina Conoci[5], Éva Fenyvesi[1], Lajos Szente[1], Milo Malanga*[1] and Salvatore Sortino*[3]

Address: [1]CycloLab, Cyclodextrin R&D Ltd, Budapest, H-1097 Illatos út 7, Hungary, [2]Department of Organic Chemistry, Faculty of Science, Charles University in Prague, Hlavová 8, 128 43, Prague 2, Czech Republic, [3]Laboratory of Photochemistry, Department of Drug Sciences, University of Catania, I-95125 Viale A. Doria 6, Italy, [4]Department of Pharmacognosy, Semmelweis University, H-1085 Üllői út 26, Hungary and [5]STMicroelectronics, Stradale Primosole 50, I-95121, Catania, Italy

Email: Milo Malanga - malanga@cyclolab.hu; Salvatore Sortino - ssortino@unict.it

*Corresponding author

Syntheses, NMR spectroscopic data, ESIMS spectra, DLS study and semi-quantitative TLC of eosin-appended β-CDs

The file contains the details of the instrumentations, the description of the procedures and the specification of the materials utilized during the study. All the spectroscopic and spectrometric data are here reported. The data collected during the aggregation study by DLS and the semi-quantitative TLCs are also shown.

Content

Instruments, general procedures and materials.

Synthesis of free dyes EoY (**2**) and EoB (**4**).

Synthesis of eosin–β-CD conjugates **2**–β-CD, **4**–β-CD.

Figure S1 ^{1}H NMR spectrum of commercially available eosin Y (source 1).

Figure S2 ^{1}H NMR spectrum of commercially available eosin Y (source 2).

Figure S3 ^{1}H NMR spectrum of eosin Y (EoY, **2**, CycloLab).

Figure S4 ^{13}C NMR spectrum of eosin Y (EoY, **2**, CycloLab).

Figure S5 COSY spectrum of eosin Y (EoY, **2**, CycloLab).

Figure S6 DEPT-ed HSQC spectrum of eosin Y (EoY, **2**, CycloLab).

Figure S7 HMBC spectrum of eosin Y (EoY, **2**, CycloLab).

Figure S8 ^{1}H NMR spectrum of commercially available eosin B (source 1).

Figure S9 ^{1}H NMR spectrum of commercially available eosin B (source 2).

Figure S10 ^{1}H NMR spectrum of dibromofluorescein (**3**, CycloLab).

Figure S11 ^{13}C NMR spectrum of dibromofluorescein (**3**, CycloLab).

Figure S12 COSY spectrum of dibromofluorescein (**3**, CycloLab).

Figure S13 DEPT-ed HSQC spectrum of dibromofluorescein (**3**, CycloLab).

Figure S14 HMBC spectrum of dibromofluorescein (**3**, CycloLab).

Figure S15 ^{1}H NMR spectrum of eosin B (EoB, **4**, CycloLab).

Figure S16 ^{13}C NMR spectrum of eosin B (EoB, **4**, CycloLab).

Figure S17 COSY spectrum of eosin B (EoB, **4**, CycloLab).

Figure S18 DEPT-ed HSQC spectrum of eosin B (EoB, **4**, CycloLab).

Figure S19 HMBC spectrum of eosin B (EoB, **4**, CycloLab).

Figure S20: ^{1}H NMR spectrum of eosin Y–β-CD (**2**–β-CD).

Figure S21: ^{13}C NMR spectrum of eosin Y–β-CD (**2**–β-CD).

Figure S22: COSY spectrum of eosin Y–β-CD (**2**–β-CD).

Figure S23: COSY spectrum (aromatic region) of eosin Y–β-CD (**2**–β-CD).

Figure S24: DEPT-ed HSQC spectrum (CD region) of eosin Y–β-CD (**2**–β-CD).

Figure S25: DEPT-ed HSQC spectrum (aromatic region) of eosin Y–β-CD (**2**–β-CD).

Figure S26: HMBC spectrum (aromatic region) of eosin Y–β-CD (**2**–β-CD).

Figure S27: ROESY spectrum of eosin Y–β-CD (**2**–β-CD).

Figure S28: ^{1}H NMR spectrum of eosin B–β-CD (**4**–β-CD).

Figure S29: ^{13}C NMR spectrum of eosin B–β-CD (**4**–β-CD).

Figure S30: COSY spectrum of eosin B–β-CD (**4**–β-CD).
Figure S31: COSY spectrum (aromatic region) of eosin B–β-CD (**4**–β-CD).
Figure S32: DEPT-ed HSQC spectrum (CD region) of eosin B–β-CD (**4**–β-CD).
Figure S33: DEPT-ed HSQC spectrum (CD region) of eosin B–β-CD (**4**–β-CD).
Figure S34: HMBC spectrum (aromatic region) of eosin B–β-CD (**4**–β-CD).
Figure S35: ROESY spectrum of eosin B–β-CD (**4**–β-CD).
Figure S36: ROESY spectrum (aromatic region) of eosin B–β-CD (**4**–β-CD).
Figure S37: MS spectrum of eosin B–β-CD (**4**–β-CD) conjugate.
Figure S38: MS spectrum of eosin Y–β-CD (**2**–β-CD) conjugate.
Figure S39: Aggregate size distribution of eosin Y (**2**)– and eosin B (**4**)–β-CD by DLS.
Figure S40: Aggregate size distribution of eosin B–β-CD (**4**–β-CD) by nanoparticles tracking analysis.
Figure S41: TLC evaluation of the purity of commercially available eosin Y from two different providers (source 1, source 2) and of eosin Y (**2**) synthesized in CycloLab.
Figure S42: TLC evaluation of the purity of commercially available eosin B from two different providers (source 1, source 2) with eosin B (**4**) synthesized in CycloLab.
Figure S43: TLC evaluation of the purity of eosin B (**4**)– and eosin Y (**2**)–β-CD conjugates.

Instruments, general procedures and materials

^{1}H NMR spectra were acquired on a Varian VXR-600 at 600 MHz, using the residual solvent signal as internal reference. Assignments were aided by COSY, DEPT-ed-HSQC, ROESY, and HMBC experiments. Mass spectra were obtained on a Bruker ESQUIRE 3000 ES-ion trap instrument with electrospray ionization (ESI) in the negative mode. All samples were dissolved in water. Thin layer chromatography (TLC) was performed on silica gel-coated aluminium sheets DC-Alufolien Kieselgel 60 F265 (Merck, Darmstadt, Germany). Dipping in 50% H_2SO_4 with subsequent carbonization by heating with a heat gun was used for the spot detection of all CD derivatives. Semi-quantitative analysis of TLC plates was performed with the software JustQuantify Free. Silica gel 60 (0.063–0.200 mm) for preparative chromatographic purification was obtained from Merck. Slide-A-Lyzer Dialysis Casette G2, cut-off MW500 (Thermo Scientific) was used for dialyses.

^1H, ^{13}C NMR and DEPT-ed-HSQC, HMBC, COSY and ROESY spectra were recorded as solutions in DMSO-d_6 or in a DMSO-d_6/$CDCl_3$ mixture (10 mg dissolved in 0.8 mL of deuterated solvent) at 298 K. UV–vis absorption and fluorescence spectra were recorded with a Jasco V 650 spectrophotometer and a Fluorolog-2 (Model, F111) spectrofluorimeter, respectively. Fluorescence lifetimes were recorded with Fluorolog-2 (Model, F111) spectrofluorimeter equipped with a TCSPC Triple Illuminator. The samples were irradiated by a pulsed diode excitation source NanoLED at 455 nm. The kinetic was monitored at 545 nm and each solution itself was used to register the prompt at 455 nm. The system allowed the measurement of fluorescence lifetimes from 200 ps. The multiexponential fit of the fluorescence decay was obtained using the following equations: $I(t) = A1\cdot \exp(^{-t/\tau 1}) + A_2\cdot \exp(^{-t/\tau 2})$ (for the biexponential fit) and $I(t) = A_1\cdot \exp(^{-t/\tau 1}) + A_2\cdot \exp(^{-t/\tau 2}) + A_3\cdot \exp(^{-t/\tau 3})$ (for the triexponential fit). $^1O_2$ emission was registered with the same spectrofluorimeter equipped with a NIR-sensitive liquid nitrogen cooled photomultiplier, exciting the air-equilibrated samples in D_2O solution at 528 nm with the fluorimeter lamp. The fluorescence and $^1O_2$ quantum yields were calculated by using eosin Y (EoY) as a reference. The aggregate size measurements were carried out using a Malvern Zetasizer Nano ZS instrument (Malvern Instruments Ltd, United Kingdom) equipped with the manufacturer’s standard 633 nm laser source. The samples were analyzed at 25 °C having the solutions filled inside a quartz cuvette of 1.00 × 1.00 cm dimensions. The particle sizes were obtained from the autocorrelation functions recorded and processed by Zetasizer Software version 6.2. Since the scattered intensity is proportional to the diameter of the particles/aggregates on the sixth power, the intensity size distribution is weighed with respect to the larger particles. Therefore this function was used to characterize the eosin–CD conjugates solutions due to its sensitive indication of the eventual aggregates present. The aggregate formation of the eosin B (**4**)–β-CD was demonstrated as well by nanoparticles tracking analysis performed with a NanoSight NS500 (Malvern Instruments Ltd.)

Reagents and chemicals

6-Monodeoxy-6-monoamino-β-CD hydrochloride is a fine chemical product of CycloLab, fluorescein disodium salt (Flu-Na, 98.5–100.5%), 4-(4,6-dimethoxy-1,3,5-triazin-2-yl)-4-methylmorpholinium chloride (DMTMMCl, >96%), 4-methylmorpholine (NMM, 99%), *N*-bromosuccinimide (ReagentPlus, 99%), Eosin Y disodium salt (dye

content ≥85%, source 1), eosin B (dye content 90%, source 1) was obtained from Sigma–Aldrich. Eosin Yellowish, eosin Y (Lot: 10187074, source 2) and eosin B (Lot: 10184683, source 2) were received from Alfa Aesar. Ethanol, acetic acid, ethyl acetate, methanol, acetone, chloroform, sulfuric acid, nitric acid, ammonium hydroxide (25% NH_3 content) was obtained from Molar Chemicals.

Experimental

Synthesis of the free dyes eosin Y (2) and eosin B (4)

Eosin Y (EoY, 2): Fluorescein (Flu, **1**, 1.1 g, 3.3 mmol) was suspended in ethanol (50 mL) then solid *N*-bromosuccinimide (NBS) was added in two portions (2 × 0.6 g, 2 × 3.3 mmol). After the addition of the first portion, the precipitate of compound **1** disappeared as the formed dibromofluorescein (DBF) was readily soluble in ethanol. During the addition of the second portion of NBS gradually crystals of **2** were formed. In order to complete the reaction, the mixture was stirred for further 30 min at rt and then allowed to crystallize for a day. The pure product **2** was recovered by filtration on a glass filter and washing the crystals with 3 × 5 mL of ethanol. Overnight drying of the crystals at 105 °C under reduced pressure (10 mbar) in the presence of P_2O_5 and KOH yielded EoY (**2**) as a deep purple crystalline material (1.9 g, 89%).

^{1}H NMR (600 MHz, DMSO-d_6) δ (ppm): 8.076 (H4, 1H, d, *J*= 7.4 Hz), 7.555 (H5, 1H, m), 7.517 (H6, 1H, m), 7.158 (H7, 1H, d, *J*= 7.1 Hz). 6.978 (H1'-H8', 2H, s). ^{13}C NMR (150 MHz, DMSO-d_6) δ (ppm): 171.20 (C3), 156.06, 135.78, 133.43 (C4), 133.17 (1'-8'), 131.83 (C5 - C6), 131.82 (C7), 120.80, 112.79, 102.01.

Dibromofluorescein (DBF, 3): Fluorescein (**1**, 1 g, 3 mmol) was suspended in acetic acid (400 mL) and the deep red suspension was heated to 60 °C. NBS (1.3 g, 7.3 mmol) was dissolved separately in 100 mL acetic acid at room temperature and added to the heated suspension of **1**. The addition of the NBS solution caused the immediate dissolution of **1**. The reaction mixture with an intensive red color was stirred at 80 °C for additional 2 h, concentrated under reduced pressure and precipitated with H_2O. The orange precipitate was collected by filtration on a glass filter and washed with water (3 × 100 mL). The crude product was dried overnight at 105 °C under reduced pressure (10 mbar) in the presence of P_2O_5 and KOH. The targeted DBF, **3** was obtained after column chromatographic purification on direct-phase silica gel using chloroform/acetone 1:1 isocratic elution in 1.02 g (70%) yield.

^{1}H NMR (600 MHz, DMSO-d_6) δ (ppm): 8.164 (H4, 1H, d, *J*= 7.6 Hz), 7.906 (H6, 1H, m), 7.835 (H5, 1H, m), 7.373 (H7, 1H, d, *J*= 7.6 Hz), 6.836 (H2'-H7', 2H, d, *J*= 8.6 Hz), 6.733 (H1'-H8', 2H, d, *J*= 9.0 Hz). ^{13}C NMR (150 MHz, DMSO-d_6) δ (ppm): 172.55 (C3), 152.25 (C3'-C6'), 137.96 (C6), 132.67 (C5), 129.66 (C1'- C8'), 127.71 (C4), 126.94 (C7), 115.24 (C2'-C7'), 113.93 (C4'-C5').

Eosin B (EoB, 4): DBF (**3**, CycloLab, 300 mg, 0.612 mmol) was dissolved in concentrated sulfuric acid (15 mL) and cooled to 0 °C. A mixture of 0.210 mL of nitric acid and 0.600 mL of sulfuric acid was added dropwise to the reaction mixture under vigorous stirring and cooling. The progress of the reaction was monitored by TLC (EtAc/methanol/NH_3 (25%) 8:2:1). After 12 h of stirring at rt, TLC monitoring showed complete conversion of starting material and the reaction mixture was poured onto crushed ice. The formed yellow precipitate was filtered, washed with water (3 × 10 mL) and dried to constant weight at 105 °C under reduced pressure (10 mbar) in the presence of P_2O_5 and KOH. The final product, EoB **4** was obtained after drying a yellow solid material in 90% (320 mg) yield.

^{1}H NMR (600 MHz, DMSO-d_6) δ (ppm): 7.977 (H4, 1H, d, *J*= 7.3 Hz), 7.865 (H6, 1H, t, *J*=7.2 x (2) Hz), 7.756 (H5, 1H, t, *J*=7.3 x (2) Hz), 7.492 (H7, 1H, d, *J*= 7.3 Hz), 7.041 (H1'-H8', 2H, s). ^{13}C NMR (150 MHz, DMSO-d_6) δ (ppm): 171.49 (C3), 168.42, 155.73, 152.54, 138.56 (C6), 137.21, 133.59 (C5), 130.79, 127.97 (C4), 127.87 (C1'-C8'), 127.74 (C7), 109.82, 104.95.

Synthesis of eosin–β-CD conjugates, 2–β-CD, 4–β-CD

(6-Eosinyl B carboxamido-6-deoxy)-β-cyclodextrin (EoB–β-CD, 4–β-CD): Eosin B (**4**, CycloLab, 186 mg, 0.32 mmol) was dissolved in H_2O (20 mL) and *N*-methylmorpholine (NMM, 105 µL, 97 mg, 0.96 mmol), 6-monodeoxy-6-monoamino-β-CD hydrochloride (374 mg, 0.32 mmol), and DMTMMCl (88 mg, 0.32 mmol) were added in sequence to the orange-colored solution. The mixture was stirred at rt for 12 h, concentrated under reduced pressure to about half of the volume and precipitated with acetone (100 mL). The solid was filtered and washed with acetone (3 × 5 mL) to remove the unreacted dye. The crude product (350 mg) was dissolved in a minimum amount of water and purified by chromatography (eluent: CH_3CN/NH_3 (25%)/H_2O 10:5:2, 10 g of silica gel per 50 mg of crude product). The collected fractions were concentrated under reduced pressure and the product precipitated by the addition of acetone (50 mL). The orange solid was filtered, washed with acetone (3 × 2 mL) and

extensively dialyzed. The solution was concentrated on a rotary evaporator and the residue dried in a vacuum drying box at 60 °C under reduced pressure (10 mbar) in the presence of P_2O_5 and KOH. Following this procedure EoB–β-CD (**4**–β-CD) was obtained as a dark orange powder in 65% (357 mg) yield.

R_f center: 0.31 in 1,4-dioxane/NH_3 10:7. ESIMS *m*/*z* found 1695.19 $[M-H]^-$, calcd for $C_{62}H_{77}Br_2N_3O_{42}$ 1696.08; *m*/*z* found 1730.10 $[M-Cl]^-$ calcd for $C_{62}H_{77}Br_2ClN_3O_{42}$ 1731.53; *m*/*z* found 847.70 $[M-2H]^{2-}$, calcd for $(C_{62}H_{77}Br_2N_3O_{42}$ - 2H)/2. ^{1}H NMR (600 MHz, DMSO-d_6) δ (ppm): 7.771 (H1, 1H, d, *J*= 6.9 Hz), 7.531 -7.450 (H2 – H3, 2H, m), 7.443 (H5, 1H, s), 7.068 (H4, 1H, d, *J*= 6.9 Hz), 7.030 (H10, 1H, s), 5.920 – 5.520 (CD-C2/C3-OH, 14H, m), 4.855-4785 (CD-H1, 7H, m), 4.470 – 4.330 (CD-C6-OH, 6H, m), 3.855 – 2.970 (CD-H2,H3,H4,H5,H6, 42H, m). ^{13}C NMR (150 MHz, DMSO-d_6) δ (ppm): 168.28, 153.44, 152.57, 152.20, 134.50, 134.21, 132.92 (C3), 129.34, 128.36 (C2), 126.34 (C5), 123.67 (C10), 122.97 (C1), 122.90 (C4), 102.00 - 101.50 (CD-C1), 84.98, 82.25, 81.82, 81.63, 81.48, 81.07, 80.01, 73.36, 72.94, 72.56, 72.17, 71.92, 66.69, 65.47, 60.42 - 59.31 (CD-C6-unsubstituted), 42.68 (CD-C6-NH-substituted).

Unreacted 6-monoamino-β-CD content <0.1 % (w/v) based on TLC.

Free EoB content: <1 % (w/v) based on TLC.

Total CD-related impurities content <0.1 % (w/v) based on TLC.

(6-Eosinyl Y-carboxamido-6-deoxy)-*β*-Cyclodextrin (EoY–β-CD, 2–β-CD): Eosin Y (**2**, CycloLab, 600 mg, 0.93 mmol) was dissolved in H_2O (30 mL) and NMM (135 μL, 124 mg, 1.22 mmol), 6-monodeoxy-6-monoamino-β-CD free base (1 g, 0.88 mmol), and DMTMMCl (250 mg, 0.9 mmol) were added in sequence to the yellow solution. The mixture was then stirred at rt for 12 h, concentrated under reduced pressure to half of the volume and precipitated with acetone (200 mL). The solid was filtered and washed with acetone (3 × 5 mL) to remove the unreacted dye. The crude product (1.3 g) was dissolved in a minimum amount of water and purified by chromatography (eluent: CH_3CN/NH_3 (25%)/H_2O 10:3:1, 10 g of silica gel per 50 mg of crude product). The collected fractions were concentrated under reduced pressure and the product precipitated by the addition of acetone (50 mL). The solid was filtered, washed with acetone (3 × 10 mL), and extensively dialyzed. The solution was concentrated on a rotary evaporator and then dried in a vacuum drying box at 60 °C under reduced pressure (10 mbar) in the presence of P_2O_5 and KOH. Following this

procedure EoY–β-CD (**2**–β-CD) was obtained as a yellow powder in 67% (1.05 g) yield.

R_f center: 0.42 in 1,4-dioxane/NH_3 10:7. ESIMS *m/z* found 1763.09 $[M-H]^-$, calculated for $C_{62}H_{77}Br_4NO_{38}$ 1763.87; *m/z* found 881.30 $[M-2H]^{2-}$ calculated for($C_{62}H_{77}Br_4NO_{38}$ - 2H)/2. ^1H NMR (600 MHz, DMSO-d_6) δ (ppm): 7.751 (H1, 1H, d, *J*= 6.2 Hz), 7.468 (H2-H3, 2H, m), 6.963 (H4, 1H, d, *J*= 6.5 Hz), 6.351 (H5-H10, 2H, s), 5.950 – 5.505 (CD-C2/C3-OH, 14H, m), 4.870 – 4.551 (CD-H1, 7H, m), 4.510 – 4.370 (CD-C6-OH, 6H, m), 3.840 – 2.740 (CD-H2,H3,H4,H5,H6, 42H, m). ^{13}C NMR (150 MHz, DMSO-d_6) δ (ppm): 168.12, 153.64, 148.78, 148.44, 132.69 (C3), 129.50, 128.22 (C2), 126.86 (C5–C10), 123.14 (C4), 122.59 (C1), 102.02 - 99.61 (CD-C1), 85.19, 81.82, 81.50, 81.09, 79.86, 73.35, 72.96, 72.58, 72.20, 71.94, 66.50, 65.92, 60.28 - 59.40 (CD-C6-unsubstituted), 42.56 (CD-C6-NH-substituted).

Unreacted 6-monoamino-*β*-CD content <0.1 % (w/v) based on TLC.

Free EoY content <1 % (w/v) based on TLC.

Total CD-related impurities content: <0.1 % (w/v) based on TLC.

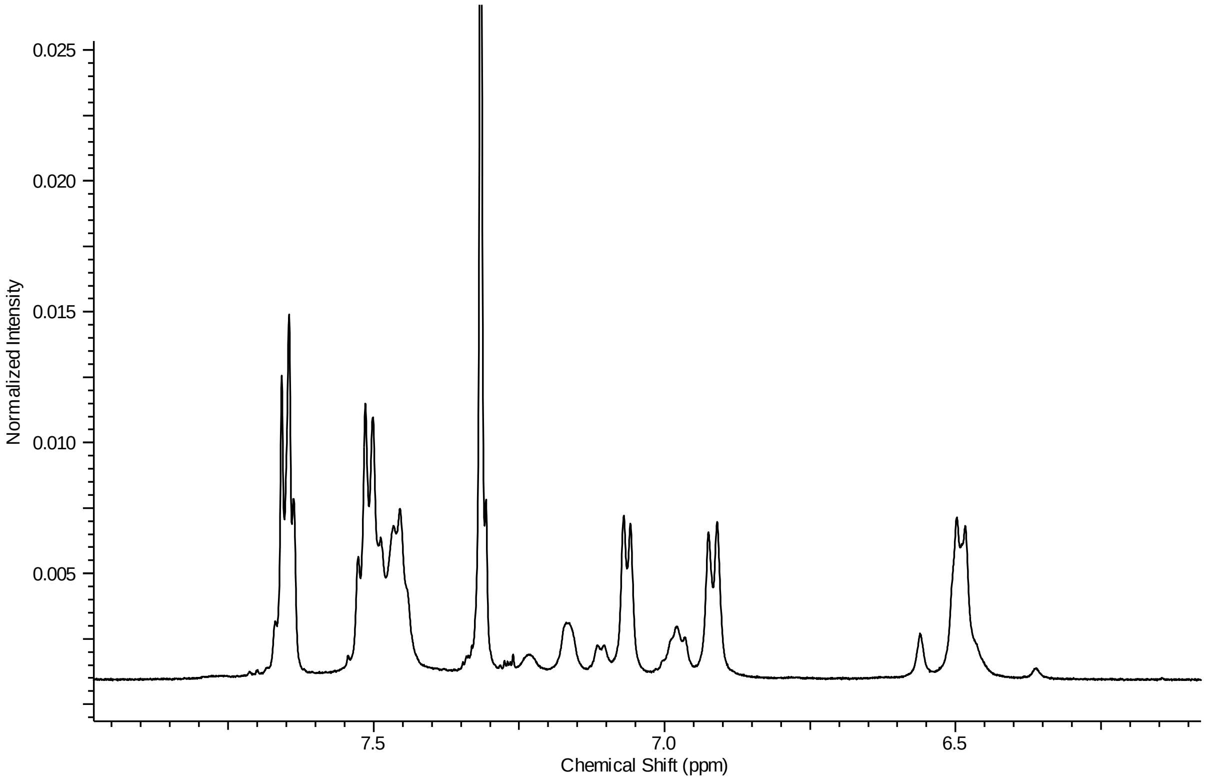

Figure S1: ^1H NMR spectrum of commercially available EoY (source 1) in D_2O (600 MHz, 298 K).

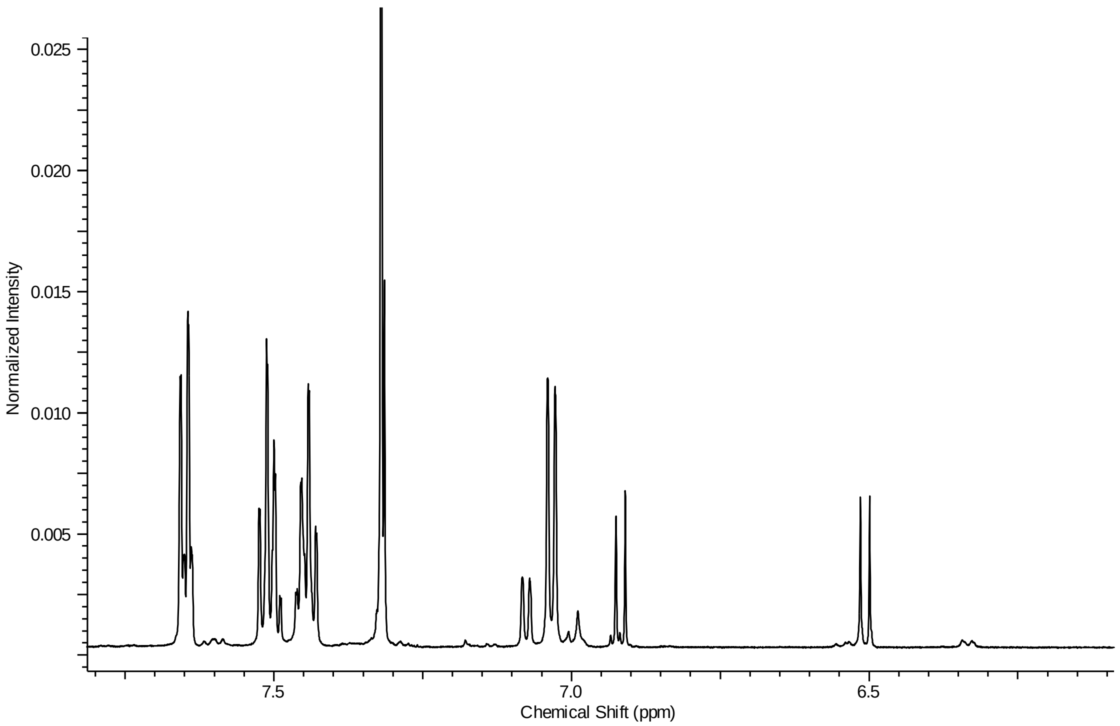

Figure S2: ^{1}H NMR spectrum of commercially available EoY (source 2) in D_2O (600 MHz, 298 K).

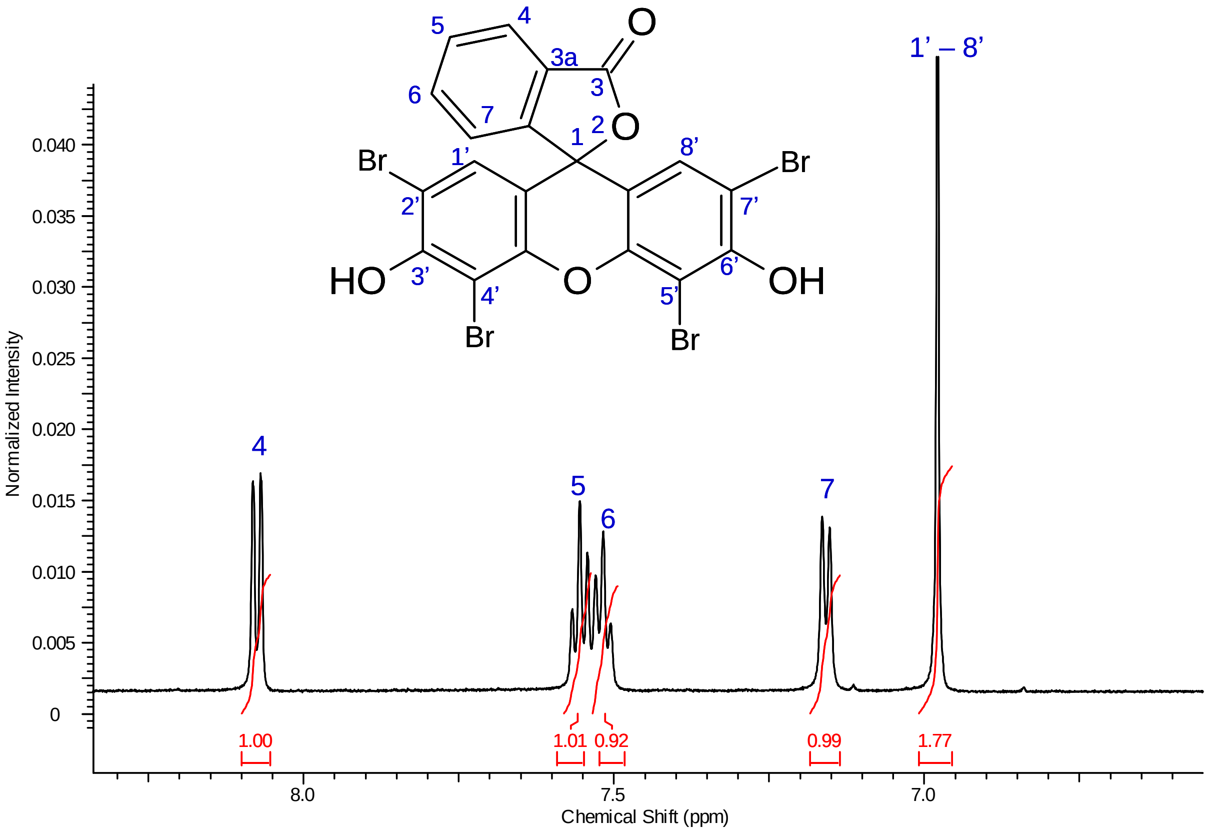

Figure S3: ^{1}H NMR spectrum of EoY (**2**, CycloLab) in DMSO-d_6 (600 MHz, 298 K).

.

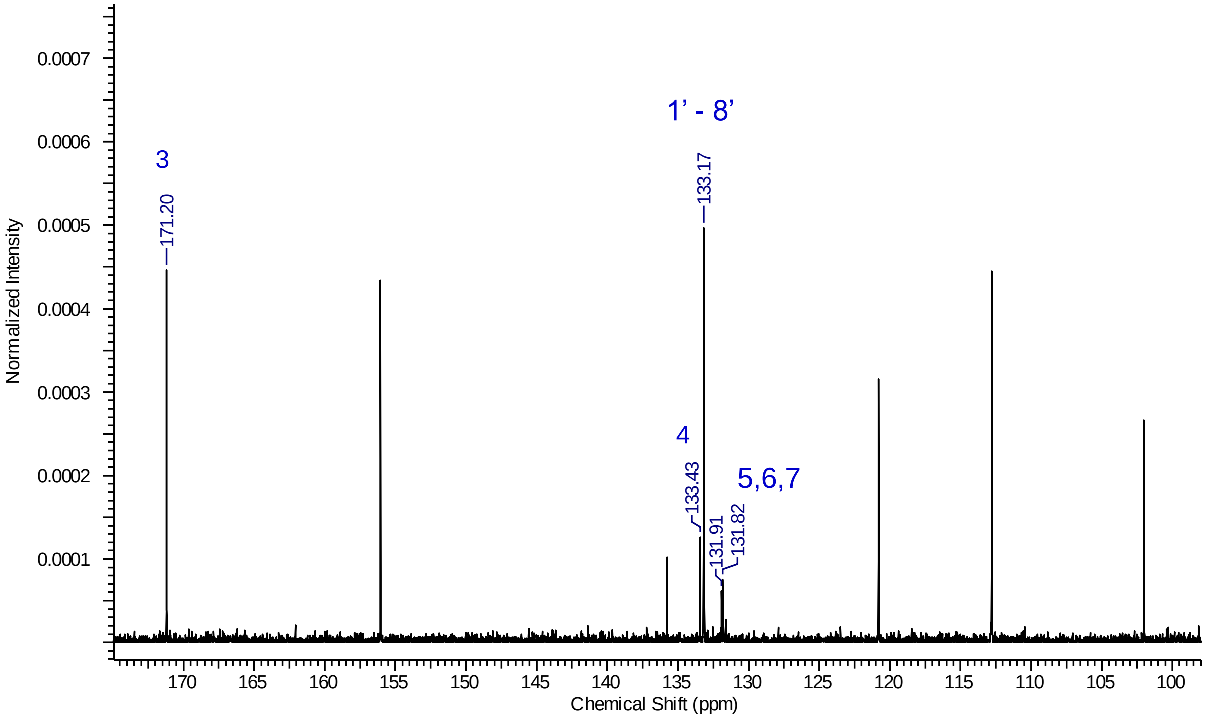

Figure S4: ^{13}C NMR spectrum of EoY (**2**, CycloLab) in DMSO-d_6 (150 MHz, 298 K).

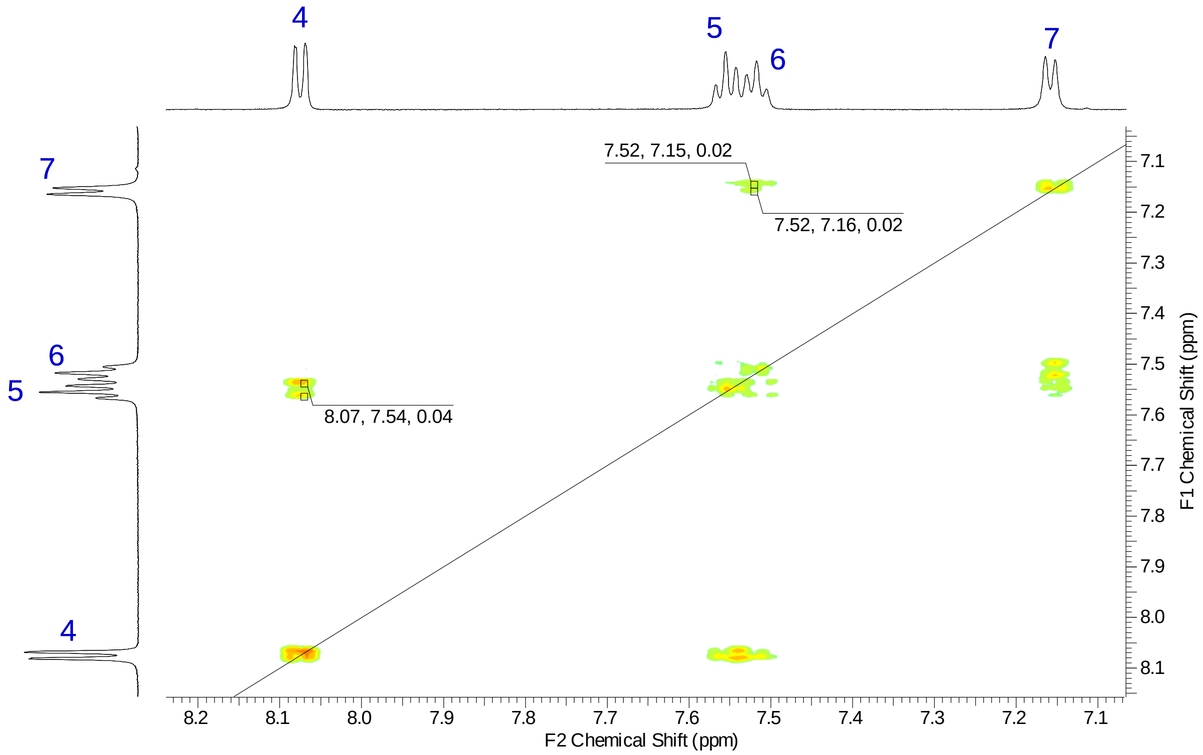

Figure S5: COSY spectrum of EoY (**2**, CycloLab) in DMSO-d_6 (600 MHz, 298 K).

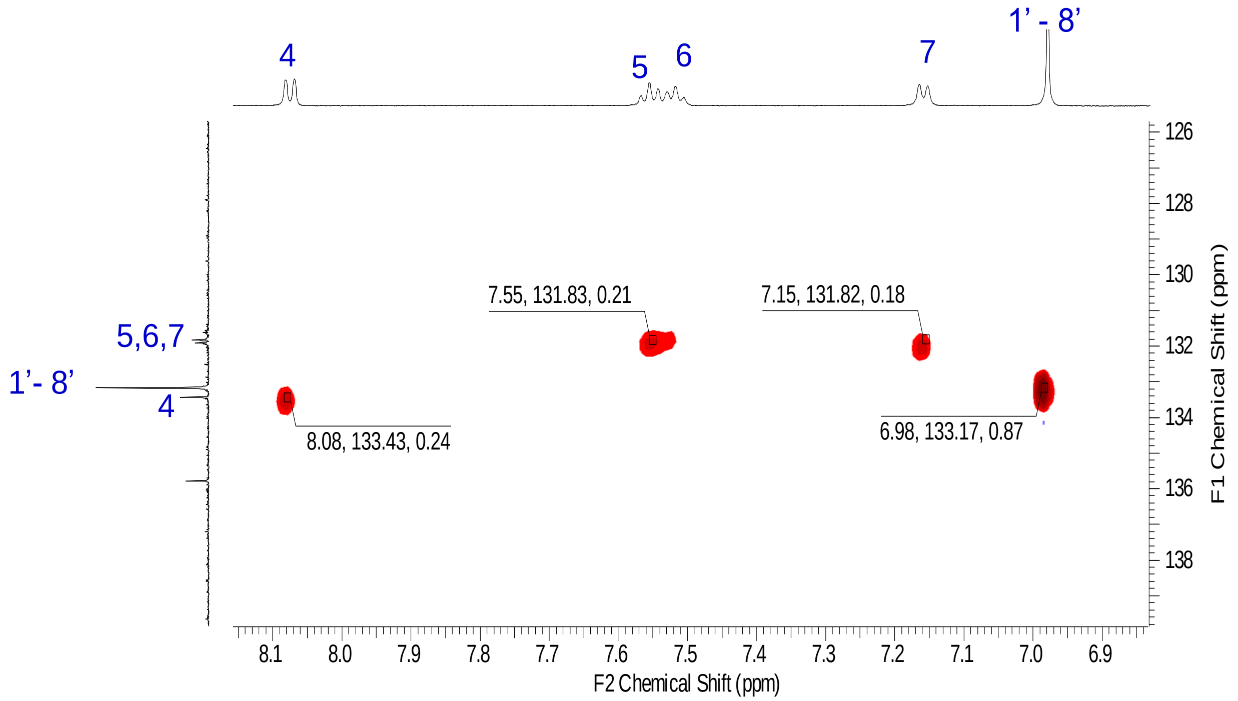

Figure S6: DEPT-ed HSQC spectrum of EoY (**2**, CycloLab) in DMSO-d_6 (600 MHz, 298 K).

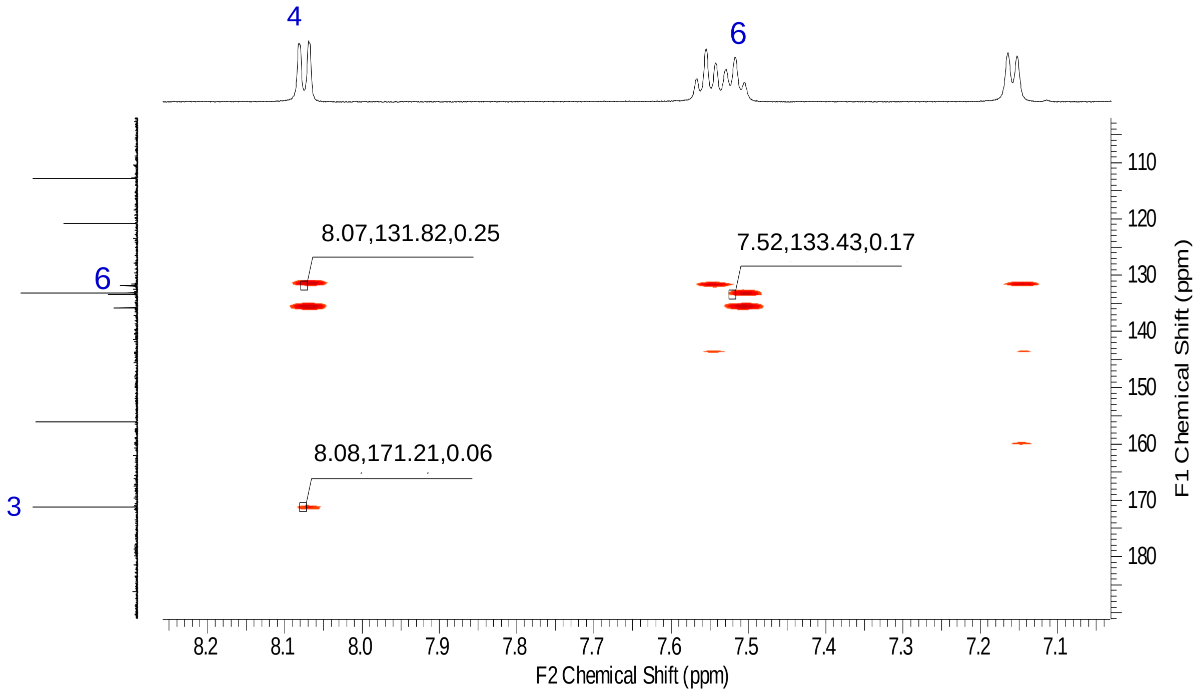

Figure S7: HMBC spectrum of EoY (**2**, CycloLab) in DMSO-d_6 (600 MHz, 298 K).

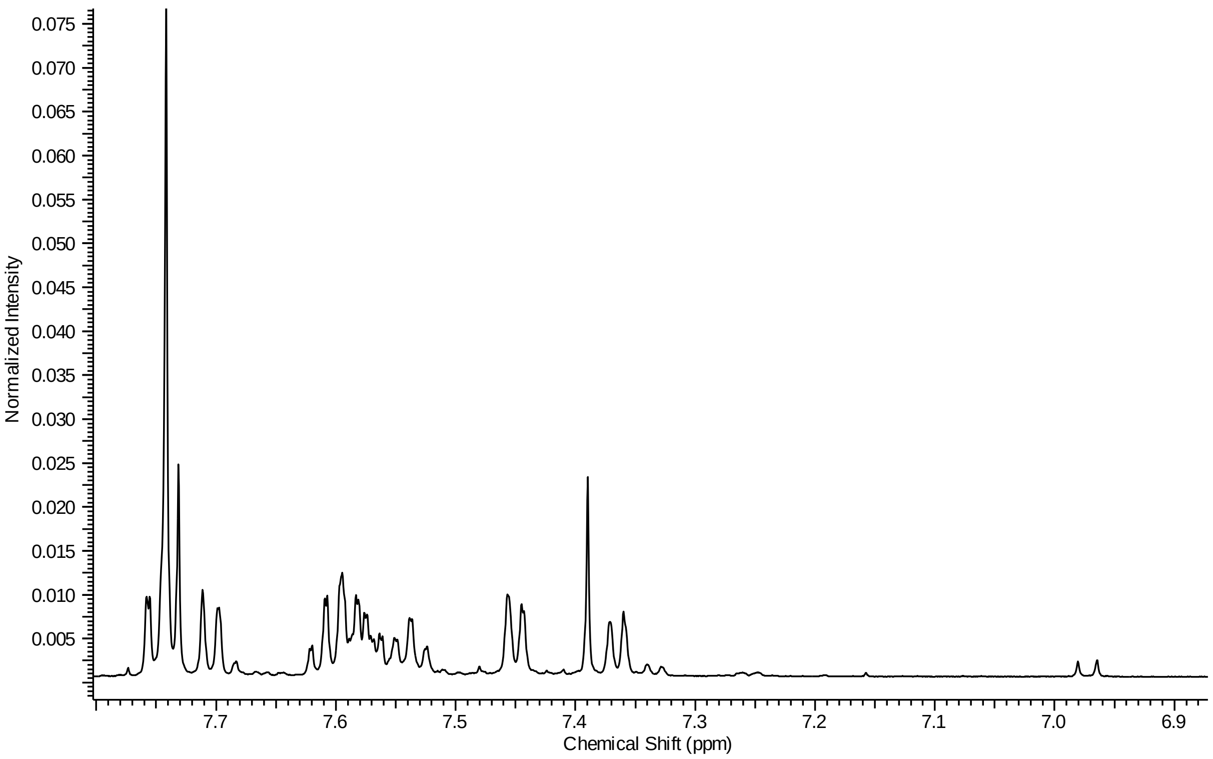

Figure S8: ^{1}H NMR spectrum of commercially available eosin B (EoB, source 1) in D_2O (600 MHz, 298 K).

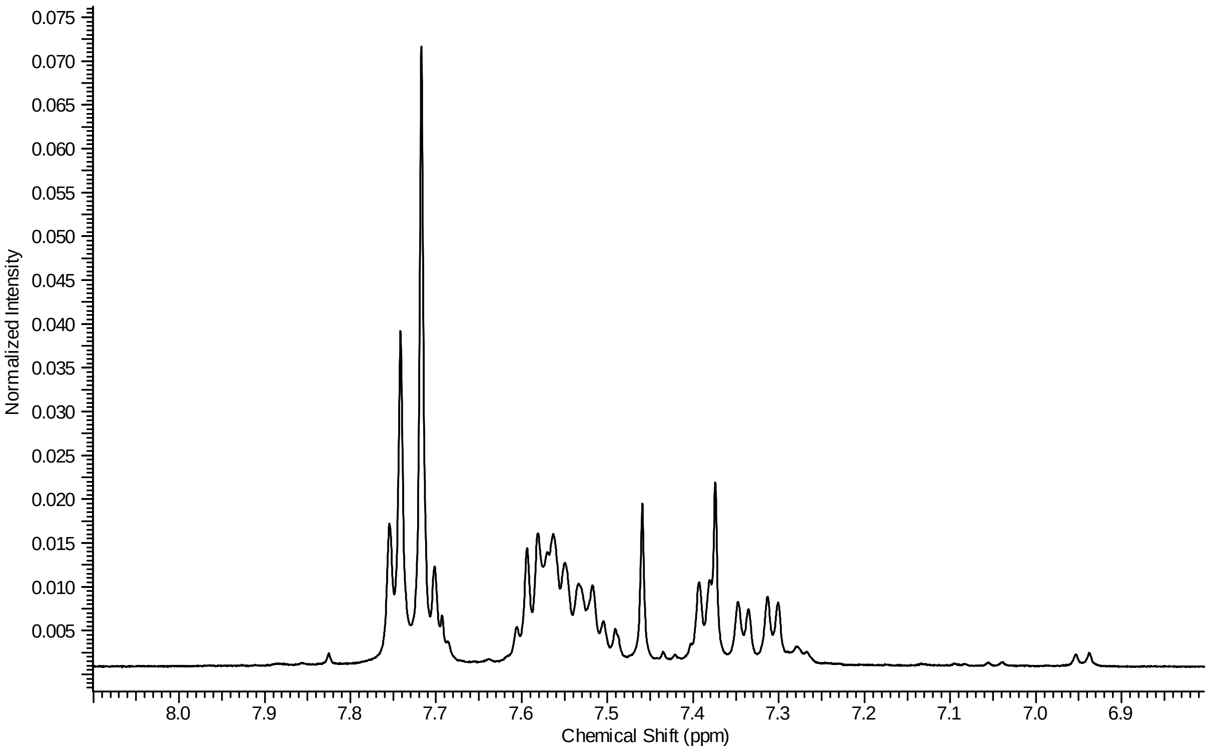

Figure S9: ^{1}H NMR spectrum of commercially available eosin B (source 2) in D_2O (600 MHz, 298 K).

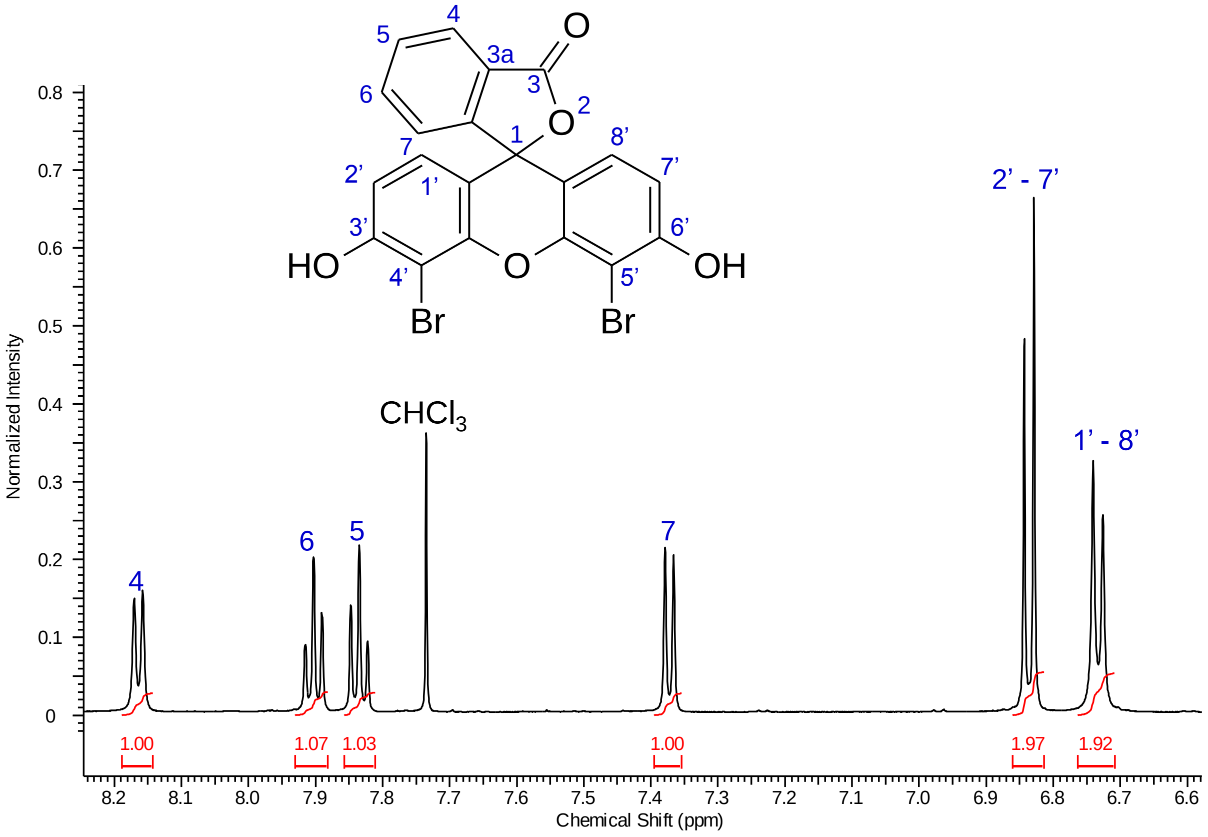

Figure S10: ^{1}H NMR spectrum of DBF (**3**, CycloLab) in DMSO-d_6 (600 MHz, 298 K).

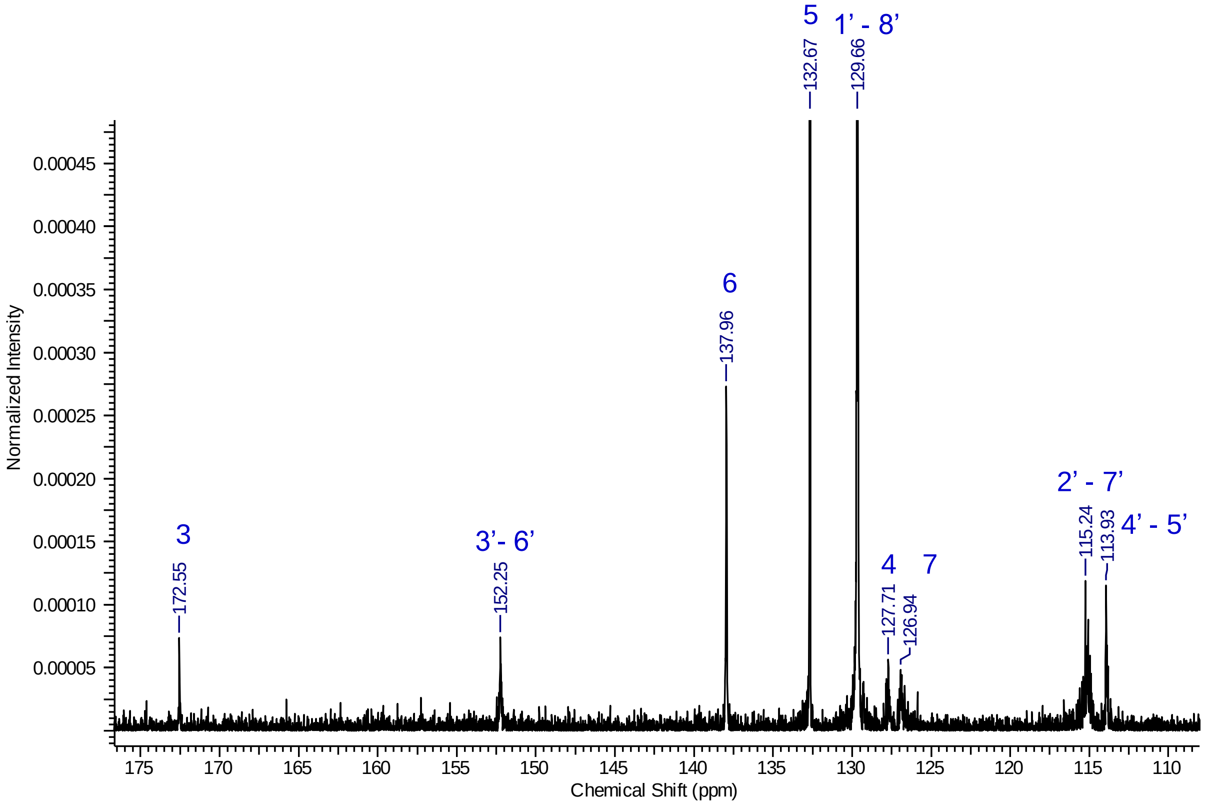

Figure S11: ^{13}C NMR spectrum of DBF (**3**, CycloLab) in DMSO-d_6 (150 MHz, 298 K).

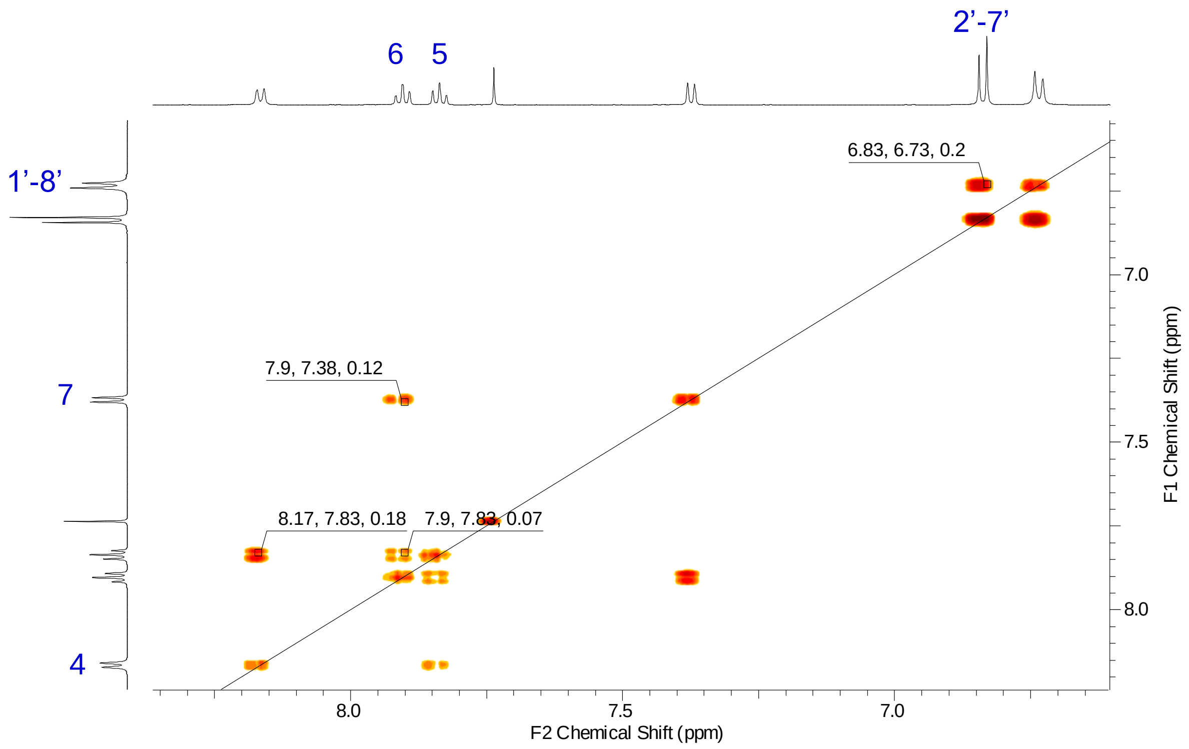

Figure S12: COSY spectrum of DBF (**3**, CycloLab) in DMSO-d_6 (600 MHz, 298 K).

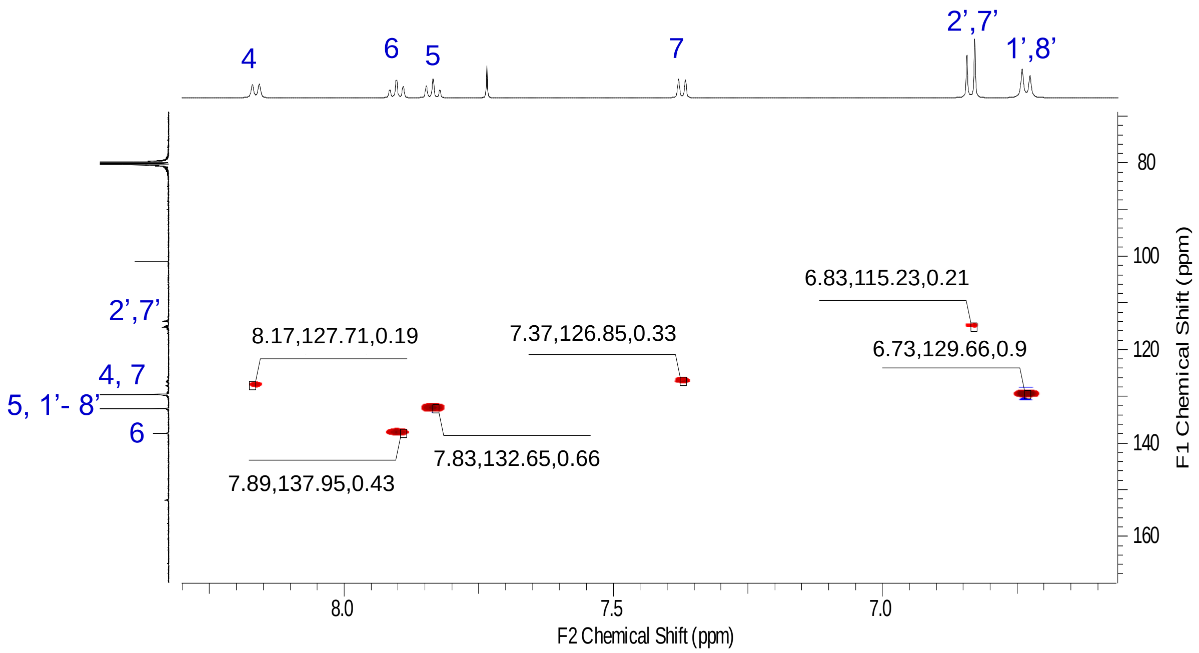

Figure S13: DEPT-ed HSQC spectrum of DBF (**3**, CycloLab) in DMSO-d_6 (600 MHz, 298 K).

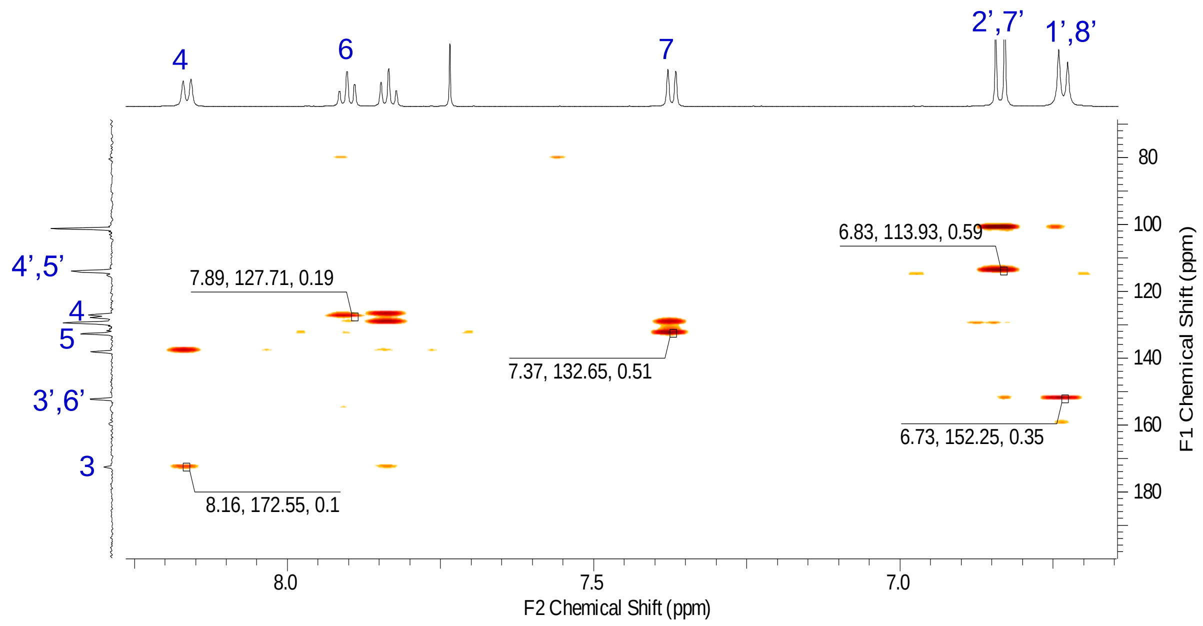

Figure S14: HMBC spectrum of DBF (**3**, CycloLab) in DMSO-d_6 (600 MHz, 298 K).

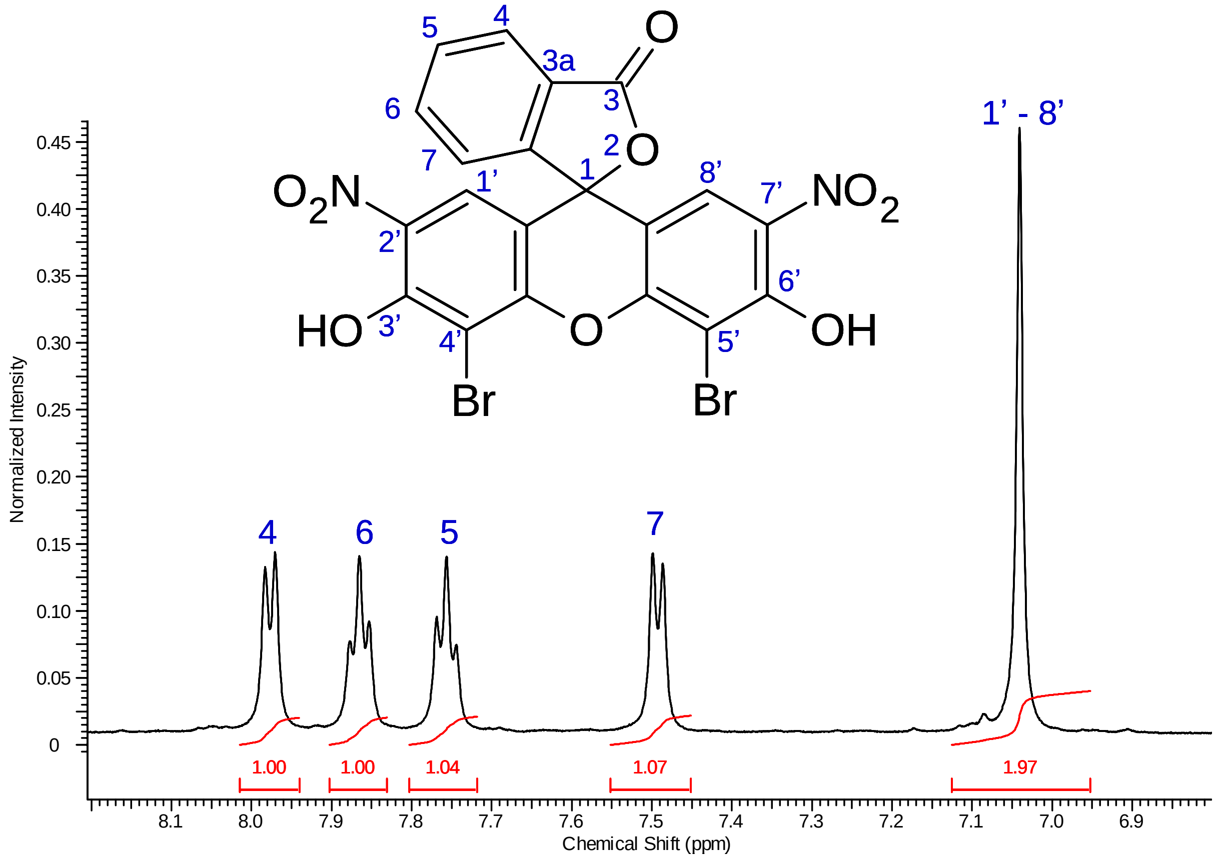

Figure S15: ^{1}H NMR spectrum of EoB (**4**, CycloLab) in DMSO-d_6 (600 MHz, 298 K).

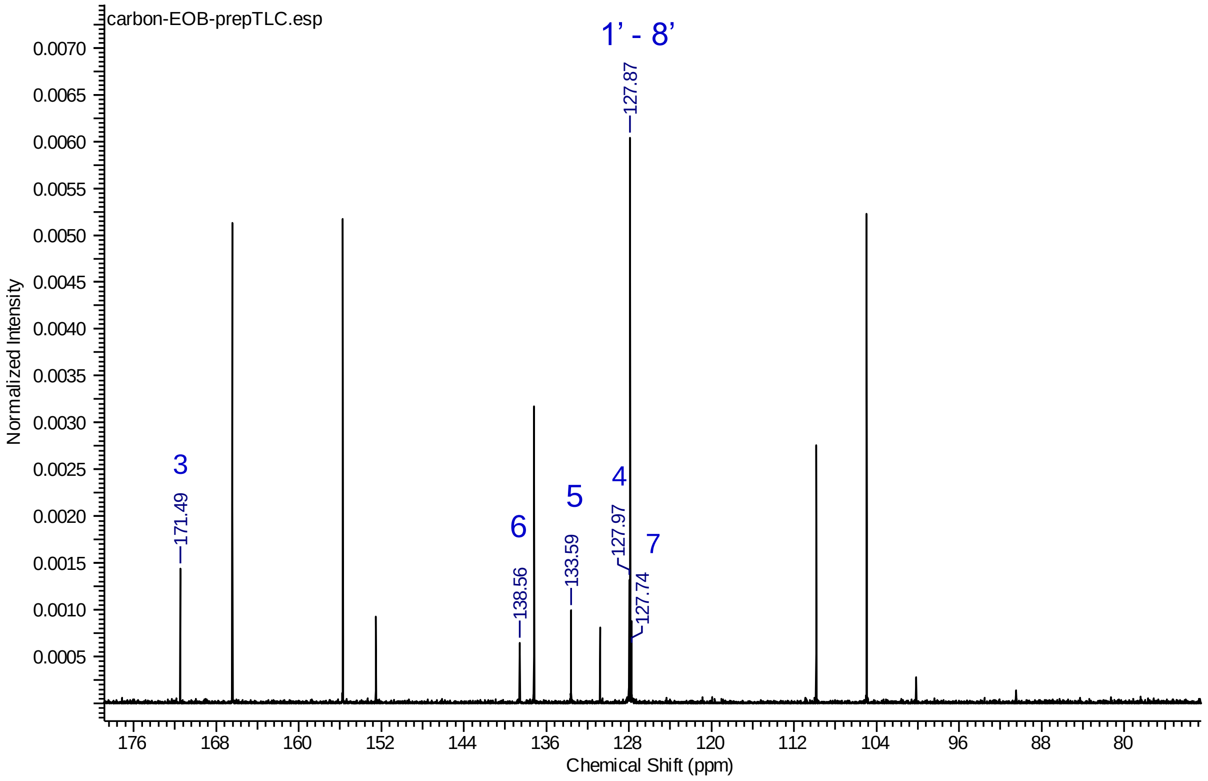

Figure S16: ^{13}C NMR spectrum of EoB (**4**, CycloLab) in DMSO-d_6 (150 MHz, 298 K).

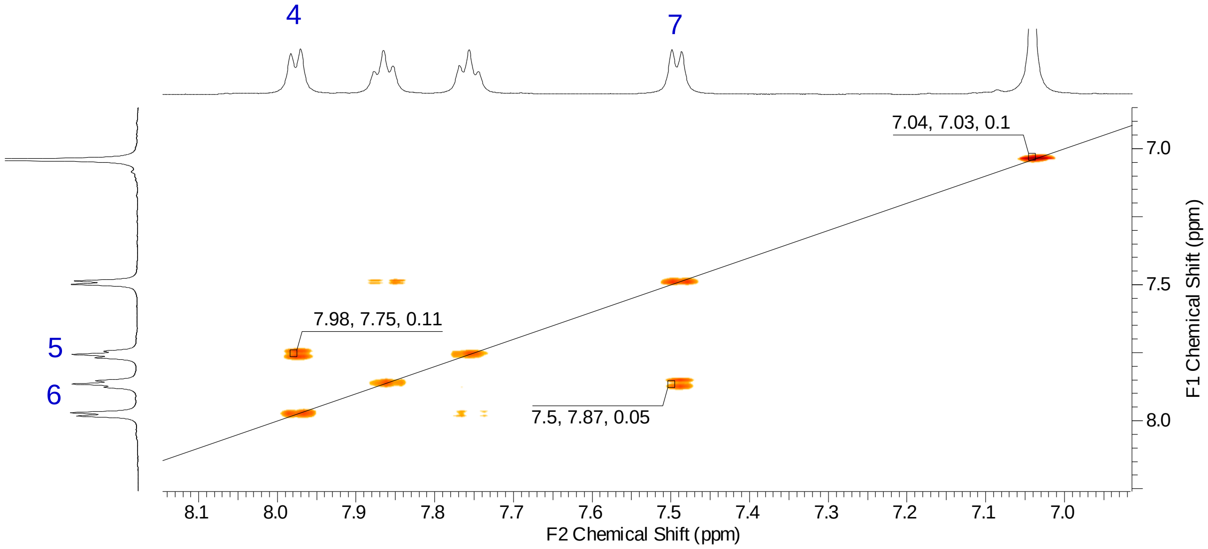

Figure S17: COSY spectrum of EoB (**4**, CycloLab) in DMSO-d_6 (600 MHz, 298 K).

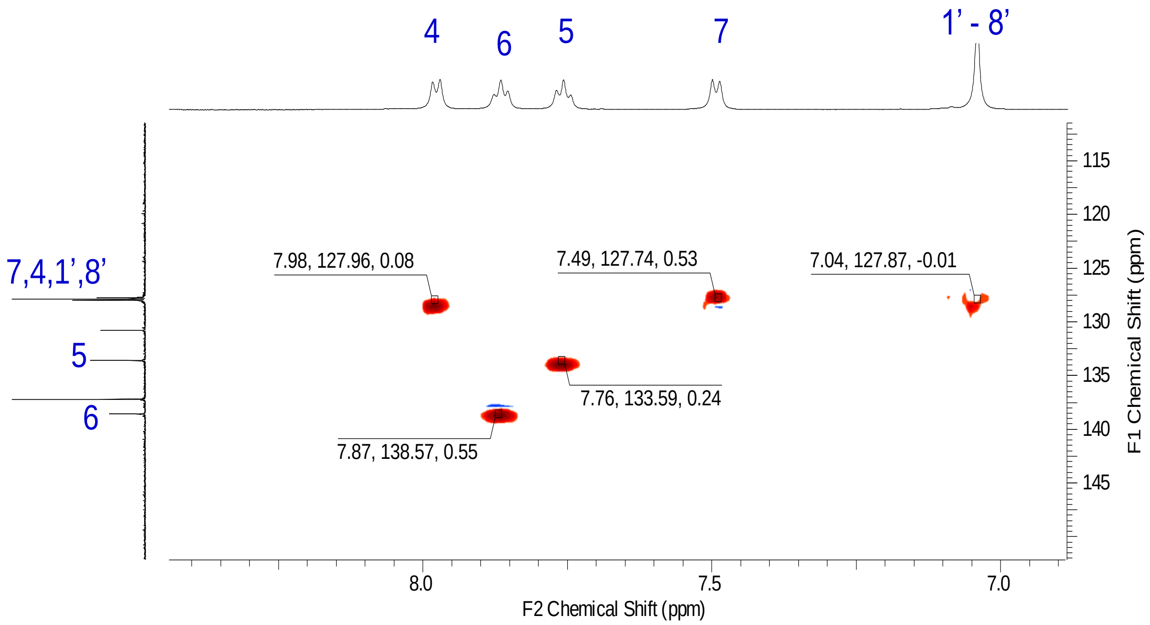

Figure S18: DEPT-ed HSQC spectrum of EoB (**4**, CycloLab) in DMSO-d_6 (600 MHz, 298 K).

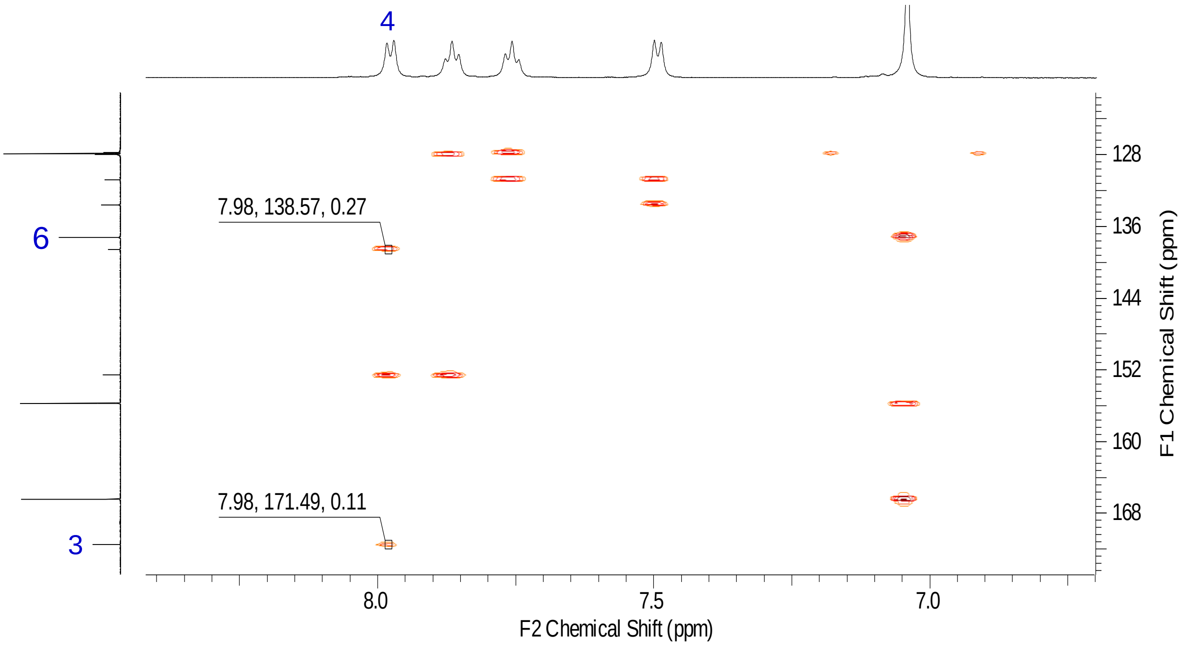

Figure S19: HMBC spectrum of EoB (**4**, CycloLab) in DMSO-d_6 (600 MHz, 298 K).

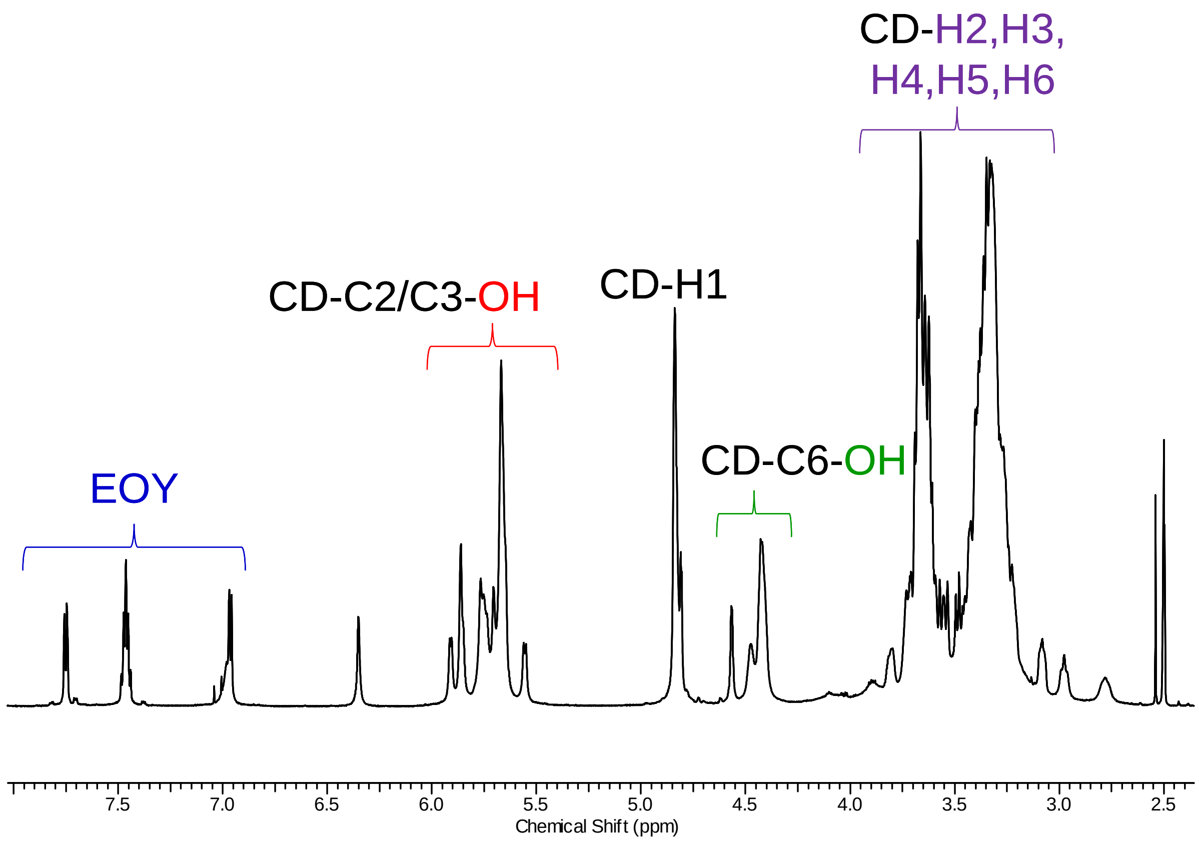

Figure S20: ^{1}H NMR spectrum of EoY–β-CD (**2**–β-CD) in DMSO-d_6 (600 MHz, 298 K).

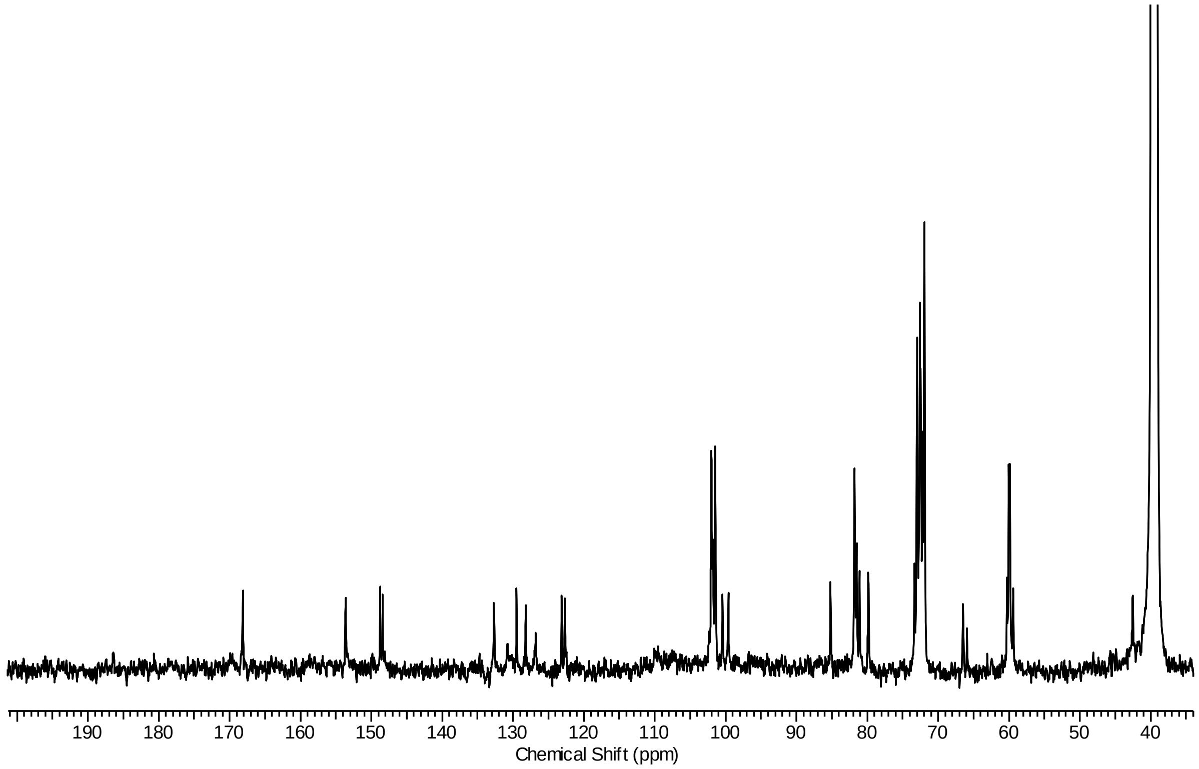

Figure S21: ^{13}C NMR spectrum of EoY–β-CD (**2**–β-CD) in DMSO-d_6 (150 MHz, 298 K).

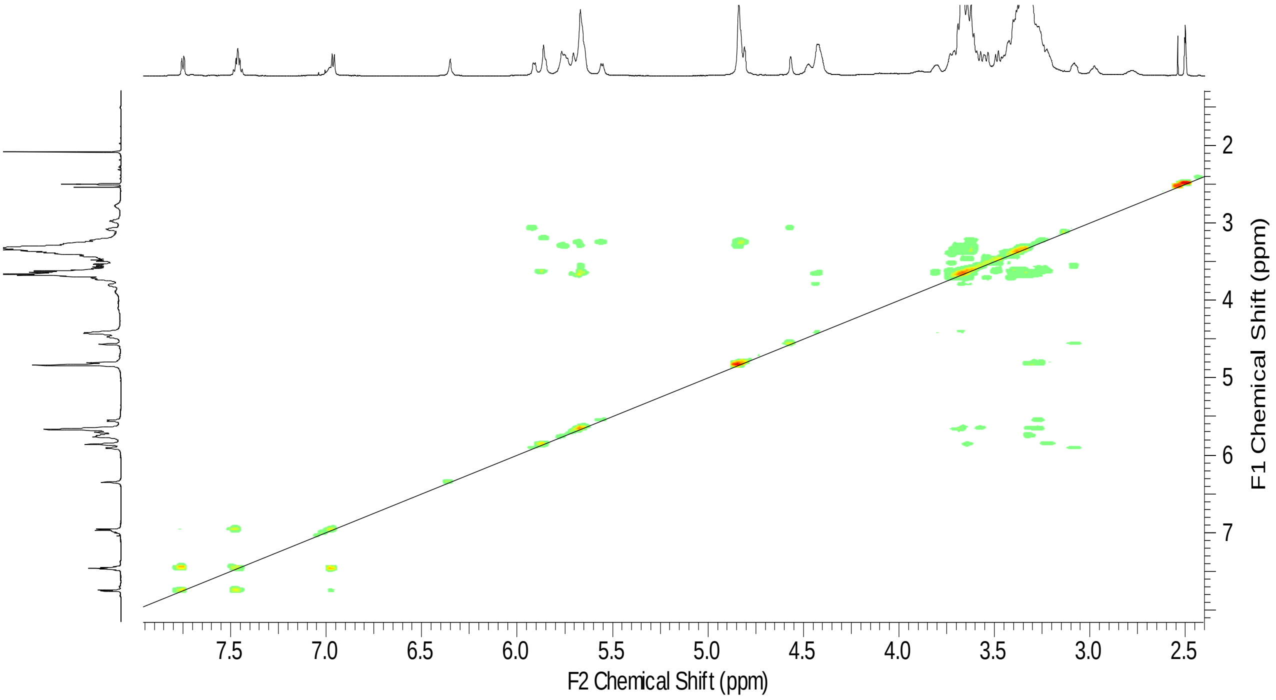

Figure S22: COSY spectrum of EoY–β-CD (**2**–β-CD) in DMSO-d_6 (600 MHz, 298 K).

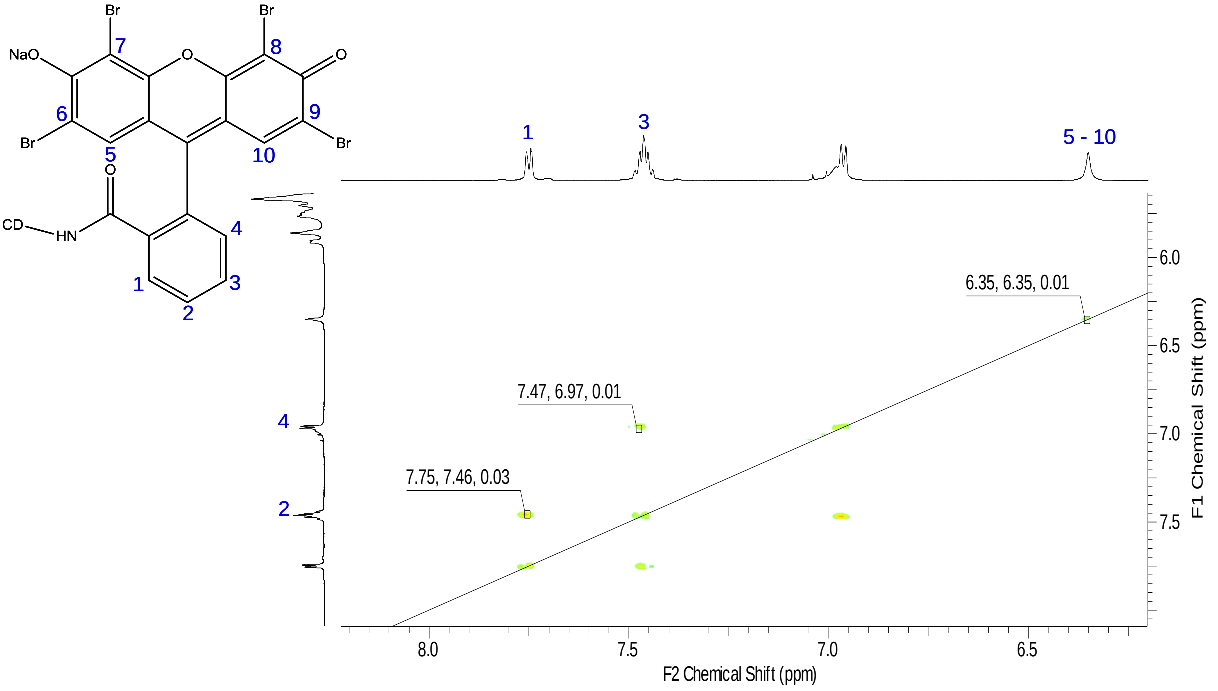

Figure S23: COSY spectrum (aromatic region) of EoY–β-CD (**2**–β-CD) in DMSO-d_6 (600 MHz, 298 K).

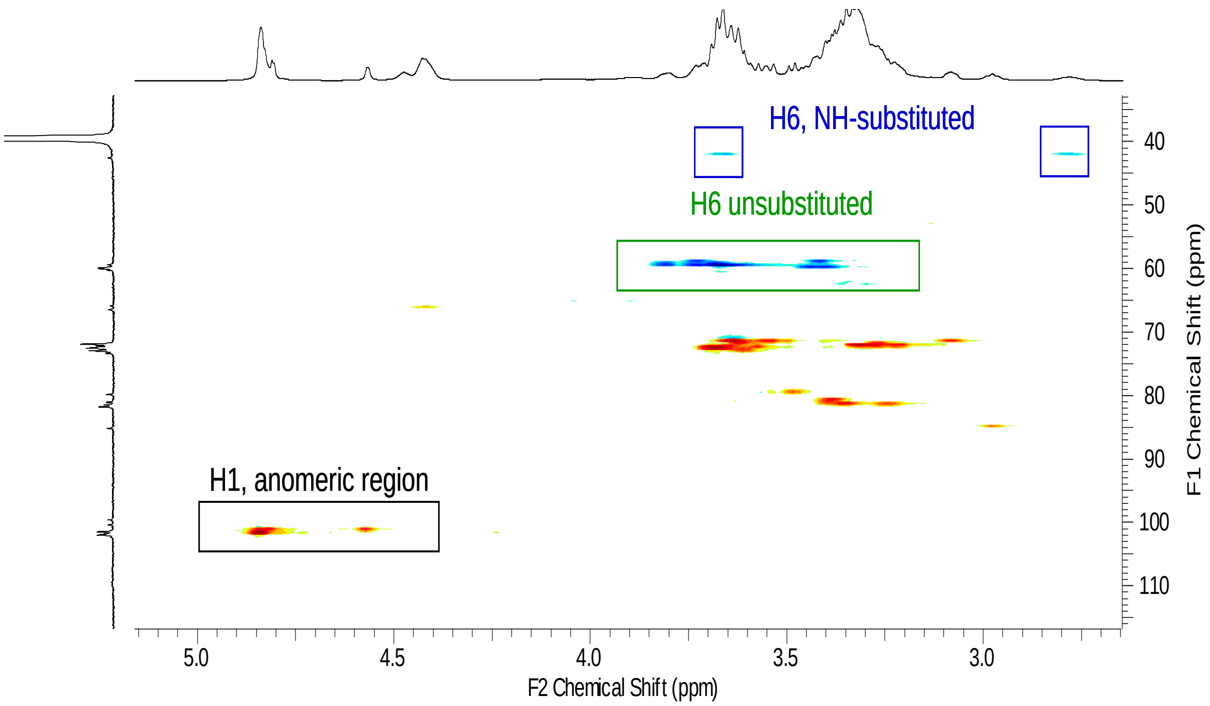

Figure S24: DEPT-ed HSQC spectrum (CD region) of EoY–β-CD (**2**–β-CD) in DMSO-d_6 (600 MHz, 298 K).

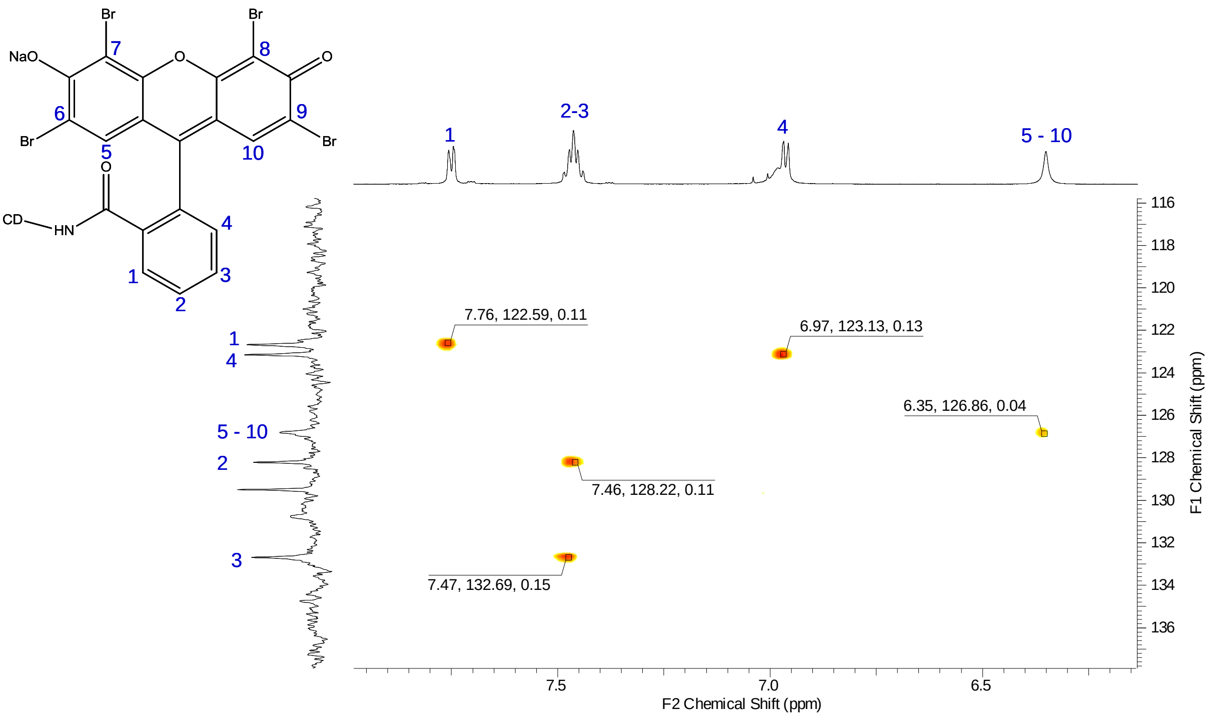

Figure S25: DEPT-ed HSQC spectrum (aromatic region) of EoY–β-CD (**2**–β-CD) in DMSO-d_6 (600 MHz, 298 K).

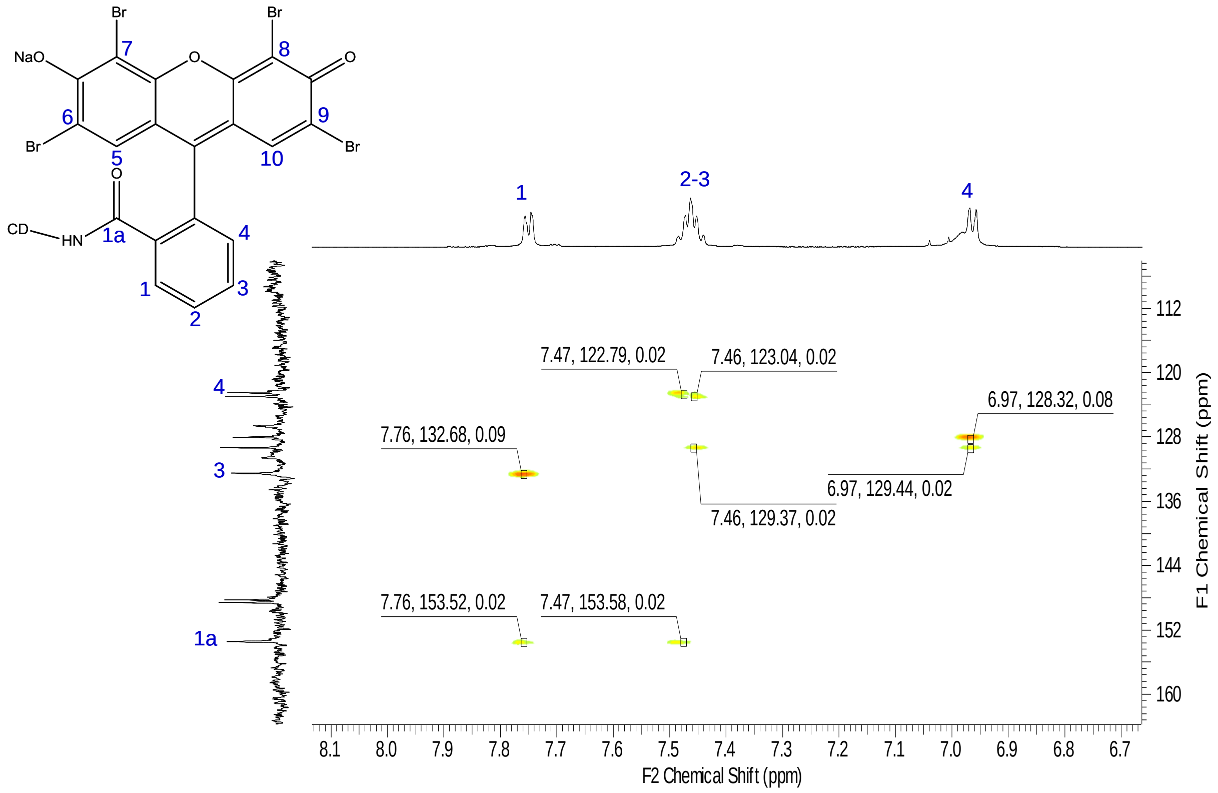

Figure S26: HMBC spectrum (aromatic region) of EoY–β-CD (**2**–β-CD) in DMSO-d_6 (600 MHz, 298 K).

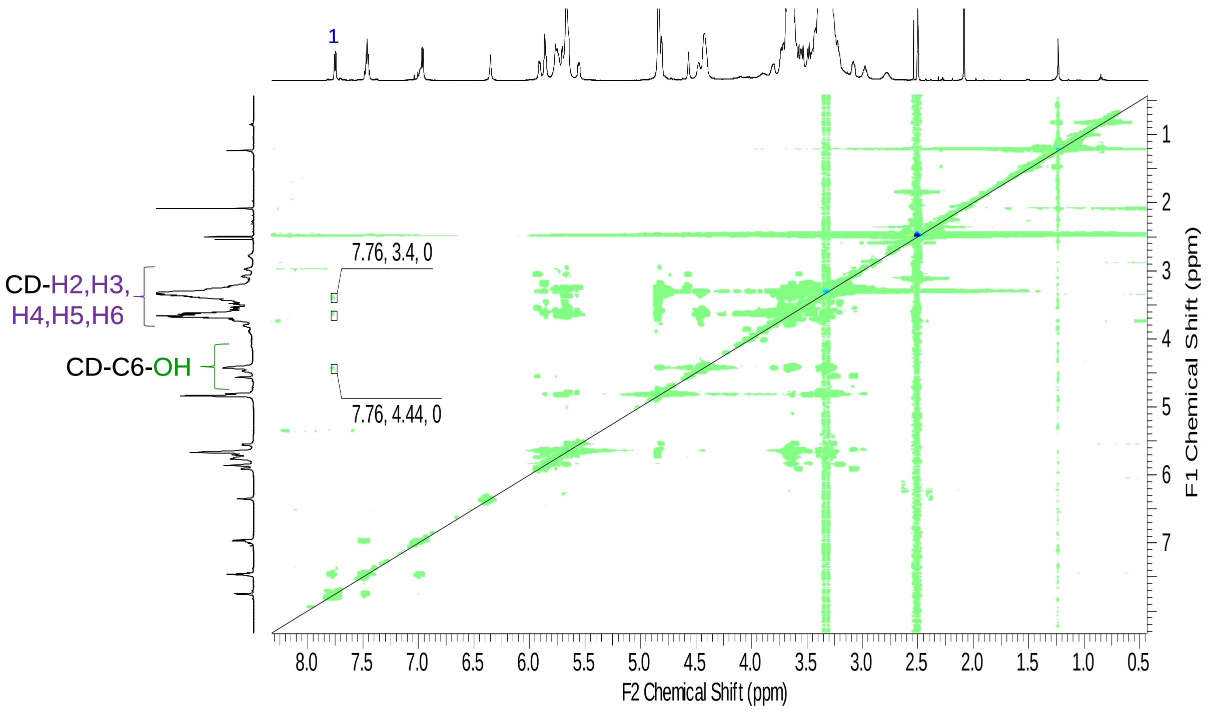

Figure S27: ROESY spectrum of EoY–β-CD (**2**–β-CD) in DMSO-d_6 (600 MHz, 298 K).

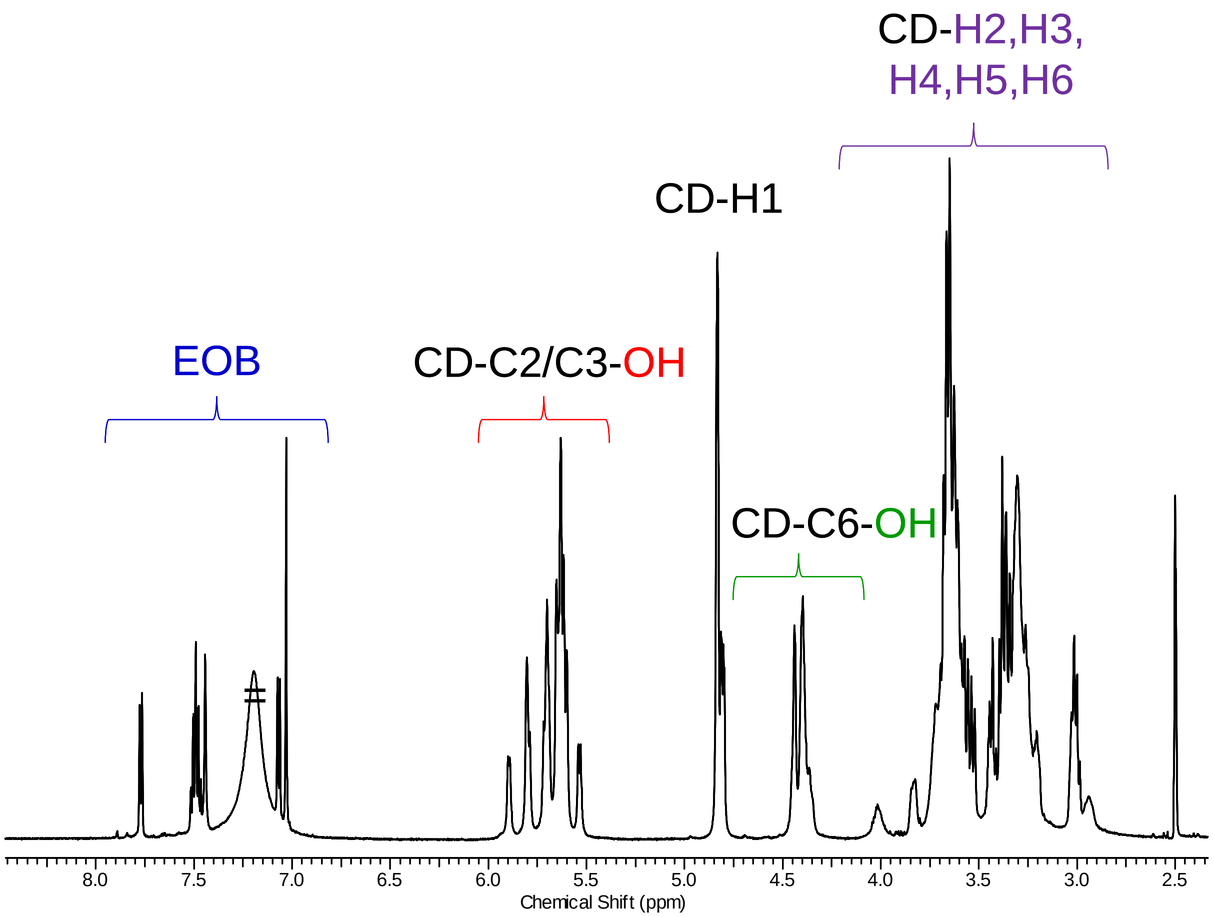

Figure S28: ^{1}H NMR spectrum of EoB–β-CD (**4**–β-CD) in DMSO-d_6 (600 MHz, 298 K).

The broad resonance at 7.2 ppm was assigned to the N-H protons of the ammonium ion. The signal is broadened due to residual coupling to ^{14}N and there is no other correlation with any other protons as is clearly shown in Figure S31 (COSY spectrum), and the lack of correlations to any carbons are confirmed by Figures S33 (DEPT-ed HSQC spectrum) and S34 (HMBC spectrum). The resonance frequency of this broad signal matches the typical frequency of the ammonium ion. Final confirmation was obtained when acidifying the solution with trifluoroacetic acid, resulting in a well-resolved 1:1:1 triplet with J = 52 Hz (due to $^1J_{14N\text{–}1H}$).

In the case of EoB–β-CD (**4**–β-CD), the dialysis process utilized for removing the ammonium salts accumulated during the chromatographic purification was only partially effective. As a consequence some part of the molecules remained as ammonium salts and are responsible for the broad peak highlighted in Figure S28.

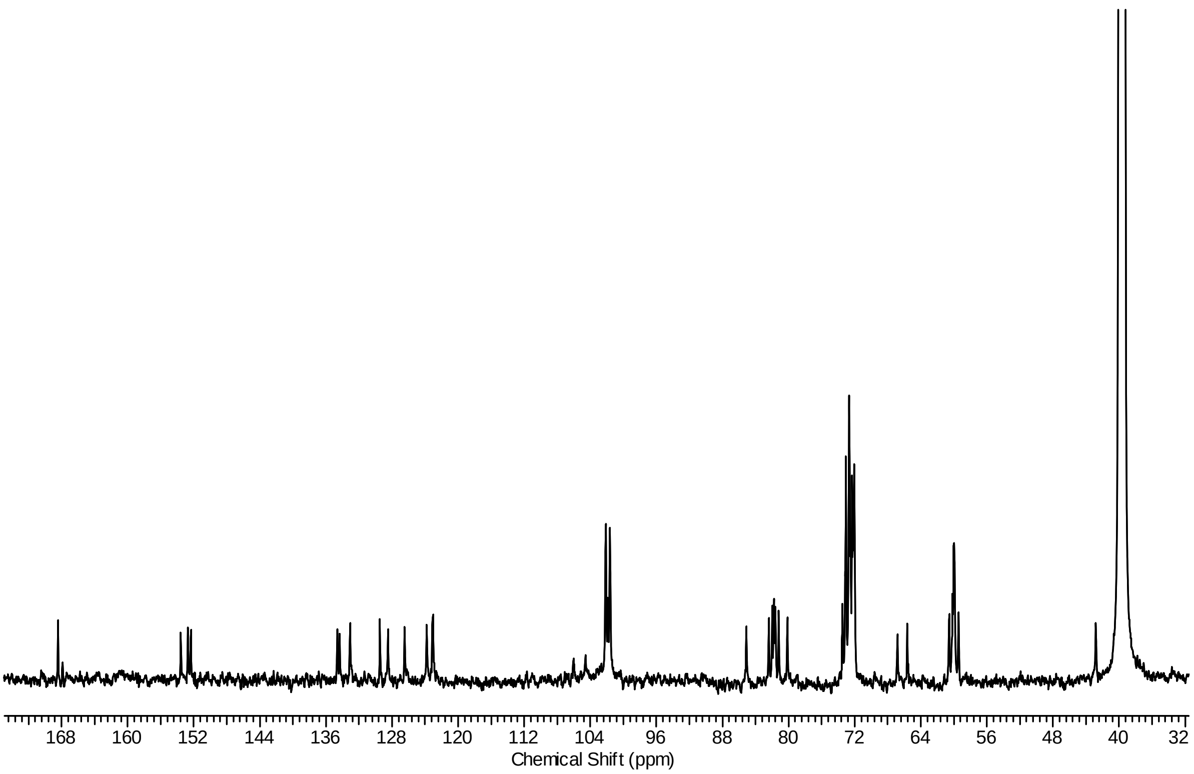

Figure S29: ^{13}C NMR spectrum of EoB–β-CD (**4**–β-CD) in DMSO-d_6 (150 MHz, 298 K).

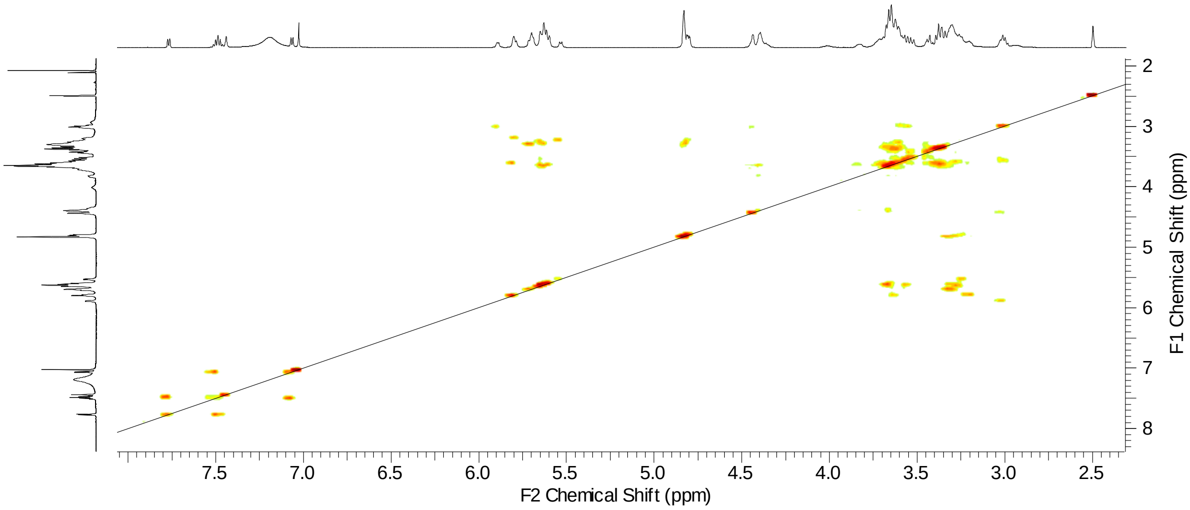

Figure S30: COSY spectrum of EoB–β-CD (**4**–β-CD) in DMSO-d_6 (600 MHz, 298 K).

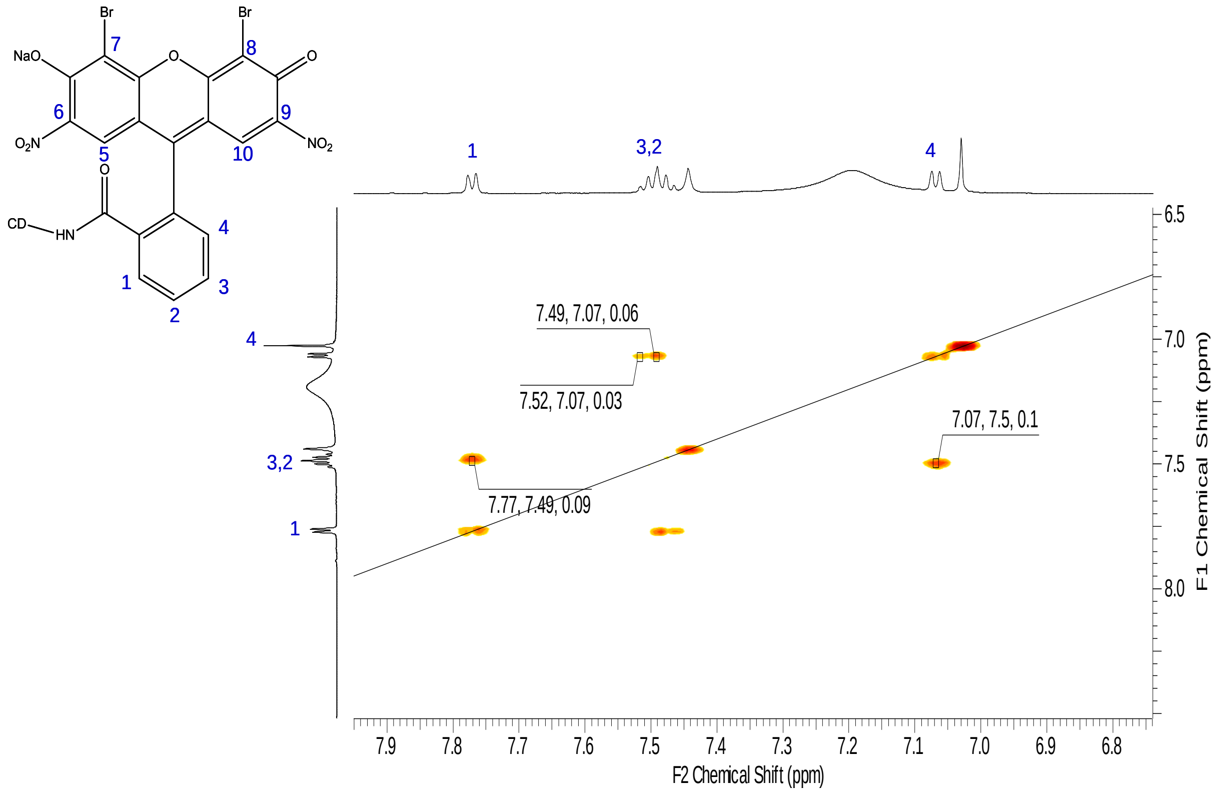

Figure S31: COSY spectrum (aromatic region) of EoB–β-CD (**4**–β-CD) in DMSO-d_6 (600 MHz, 298 K).

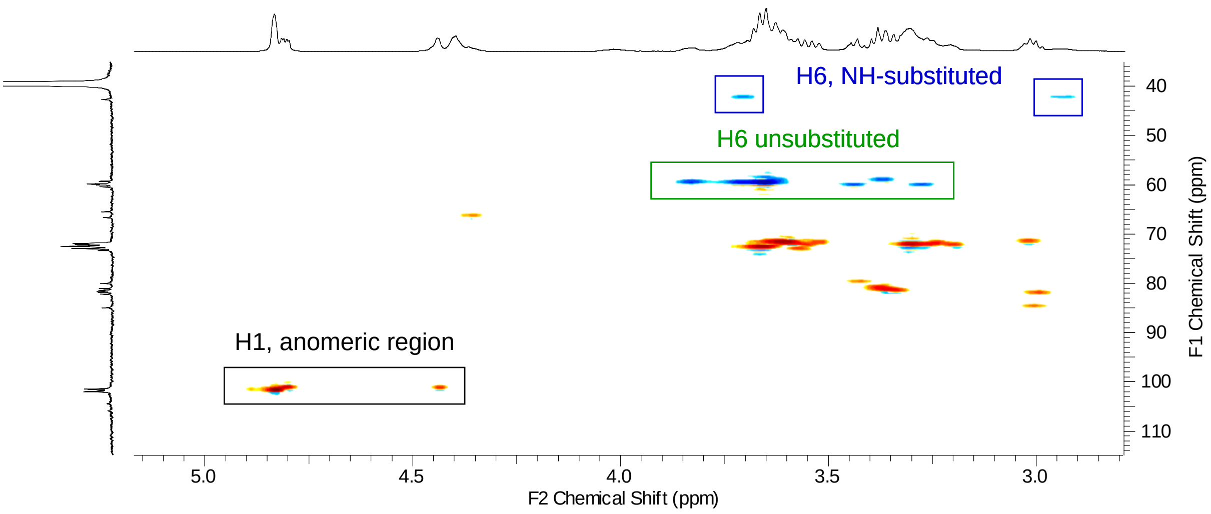

Figure S32: DEPT-ed HSQC spectrum (CD region) of EoB–β-CD (**4**–β-CD) in DMSO-d_6 (600 MHz, 298 K).

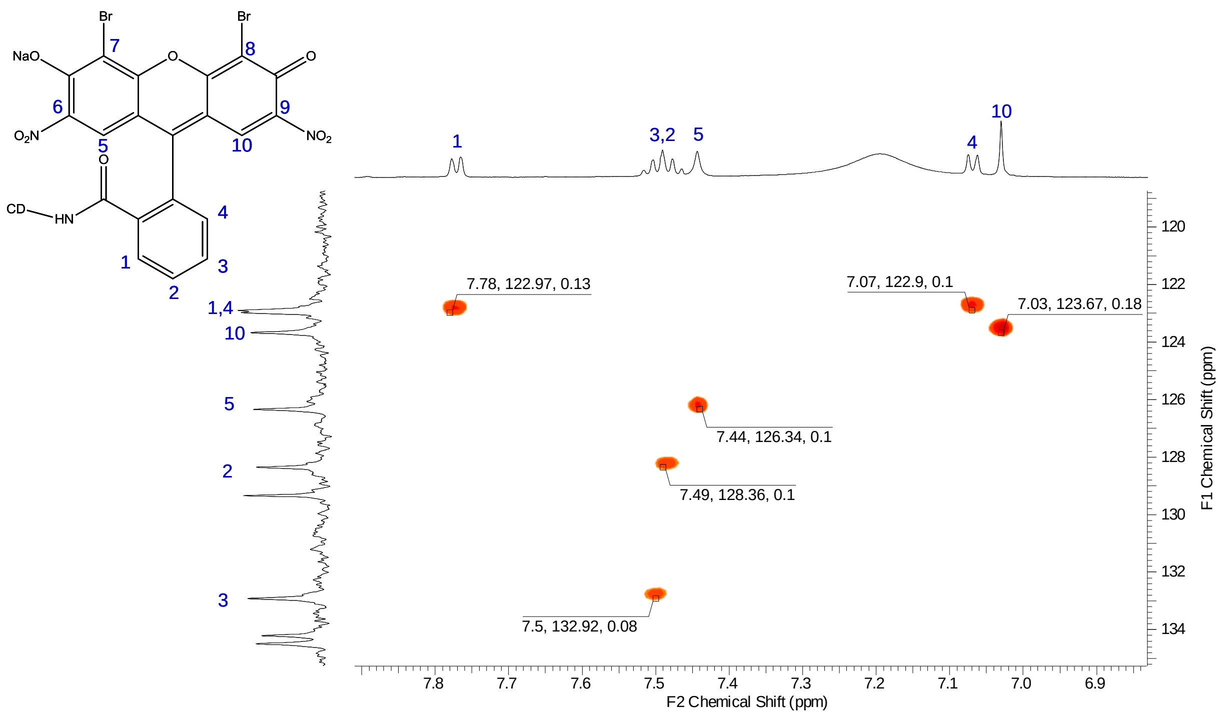

Figure S33: DEPT-ed HSQC spectrum (CD region) of EoB–β-CD (**4**–β-CD) in DMSO-d_6 (600 MHz, 298 K).

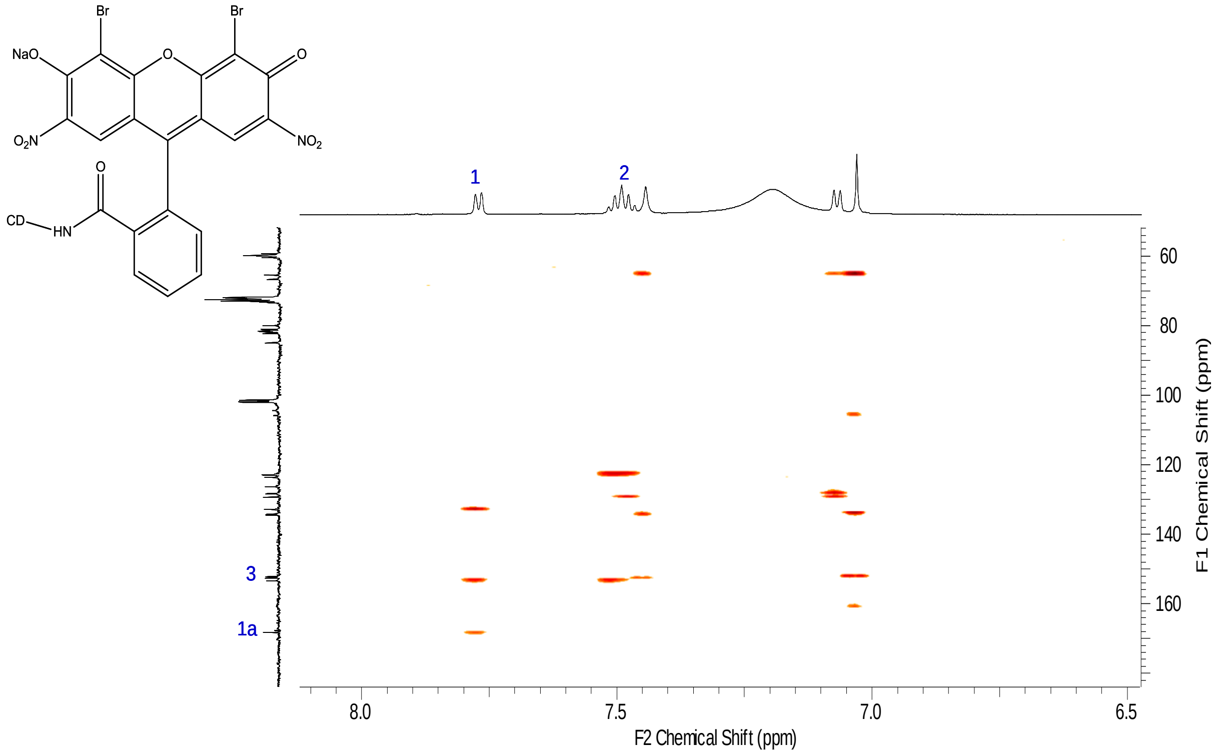

Figure S34: HMBC spectrum (aromatic region) of EoB–β-CD (**4**–β-CD) in DMSO-d_6 (600 MHz, 298 K).

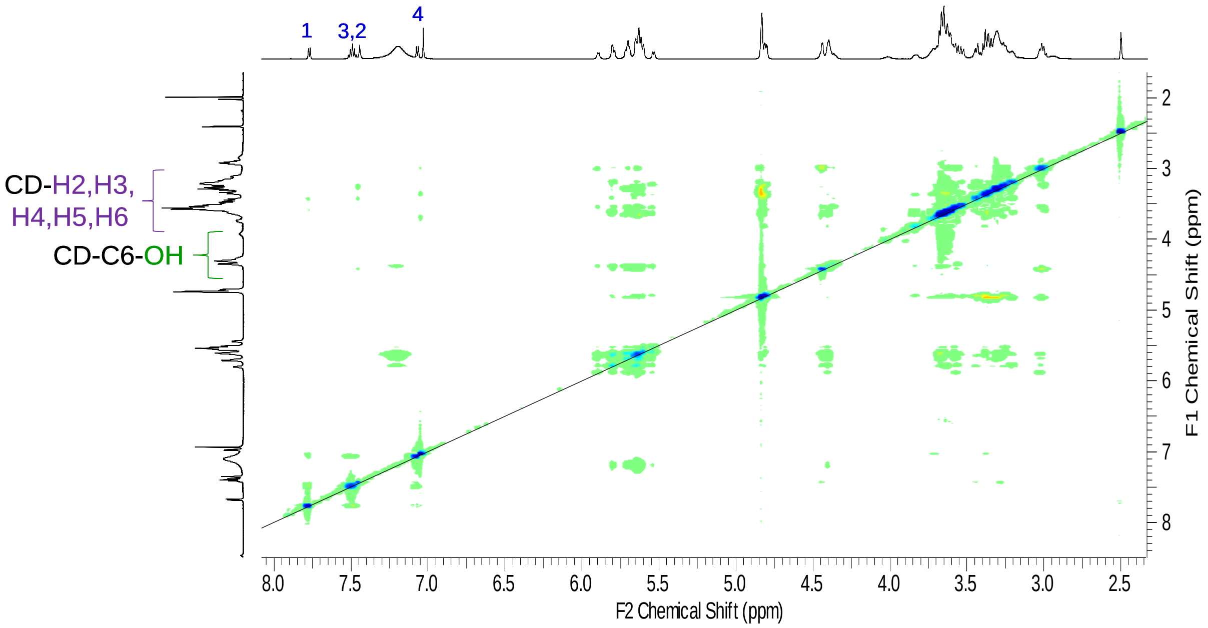

Figure S35: ROESY spectrum of EoB–β-CD (**4**–β-CD) in DMSO-d_6 (600 MHz, 298 K).

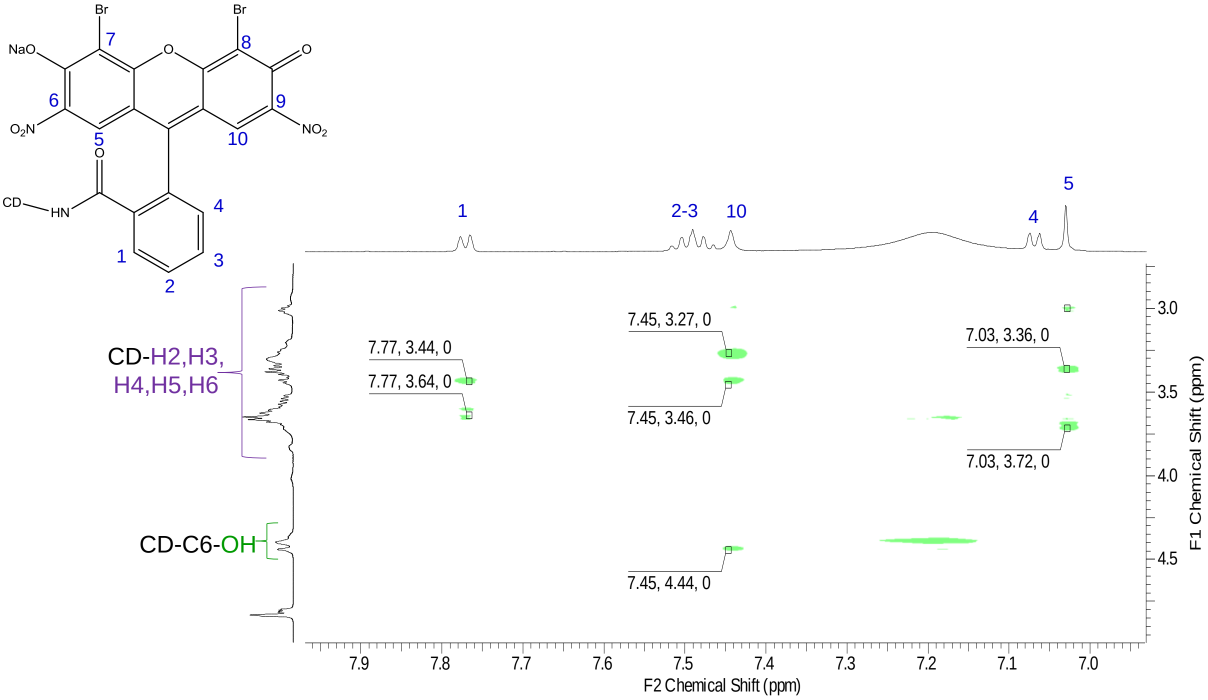

Figure S36: ROESY spectrum (aromatic region) of EoB–β-CD (**4**–β-CD) in DMSO-d_6 (600 MHz, 298 K).

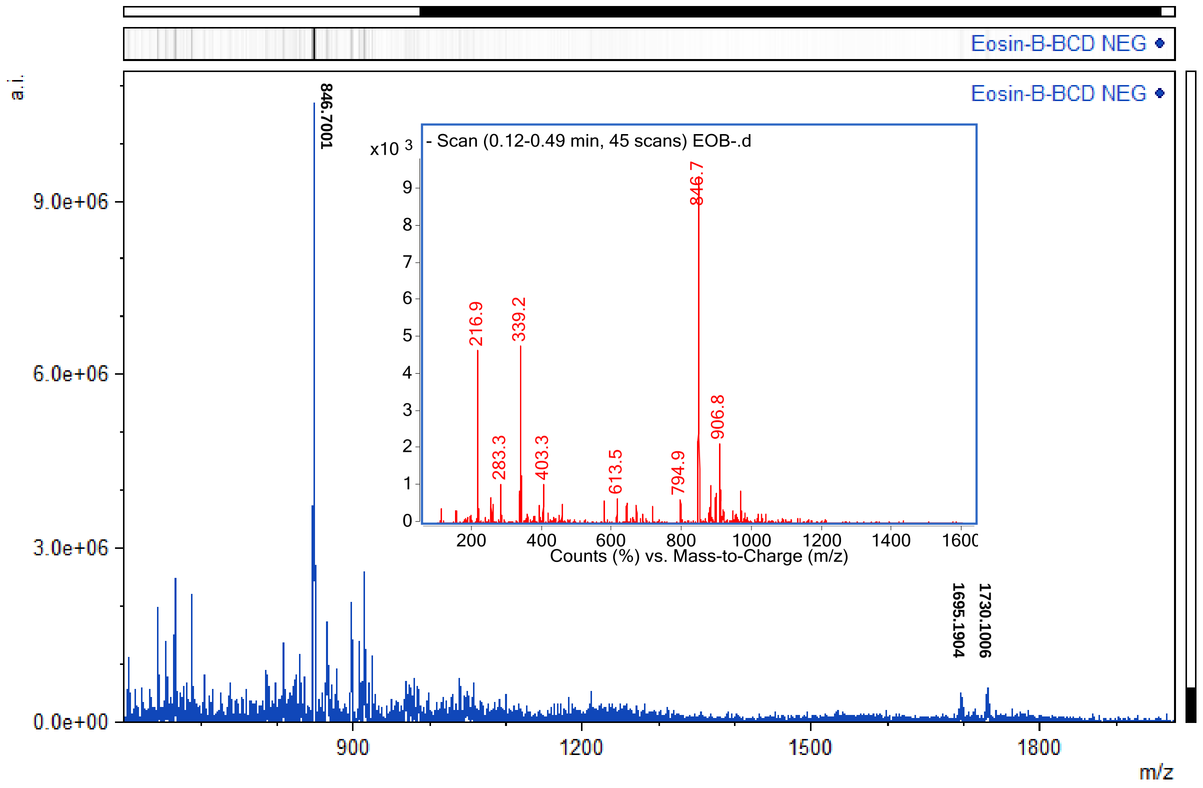

Figure S37: MS spectrum of EoB–β-CD (**4**–β-CD) conjugate in negative mode.

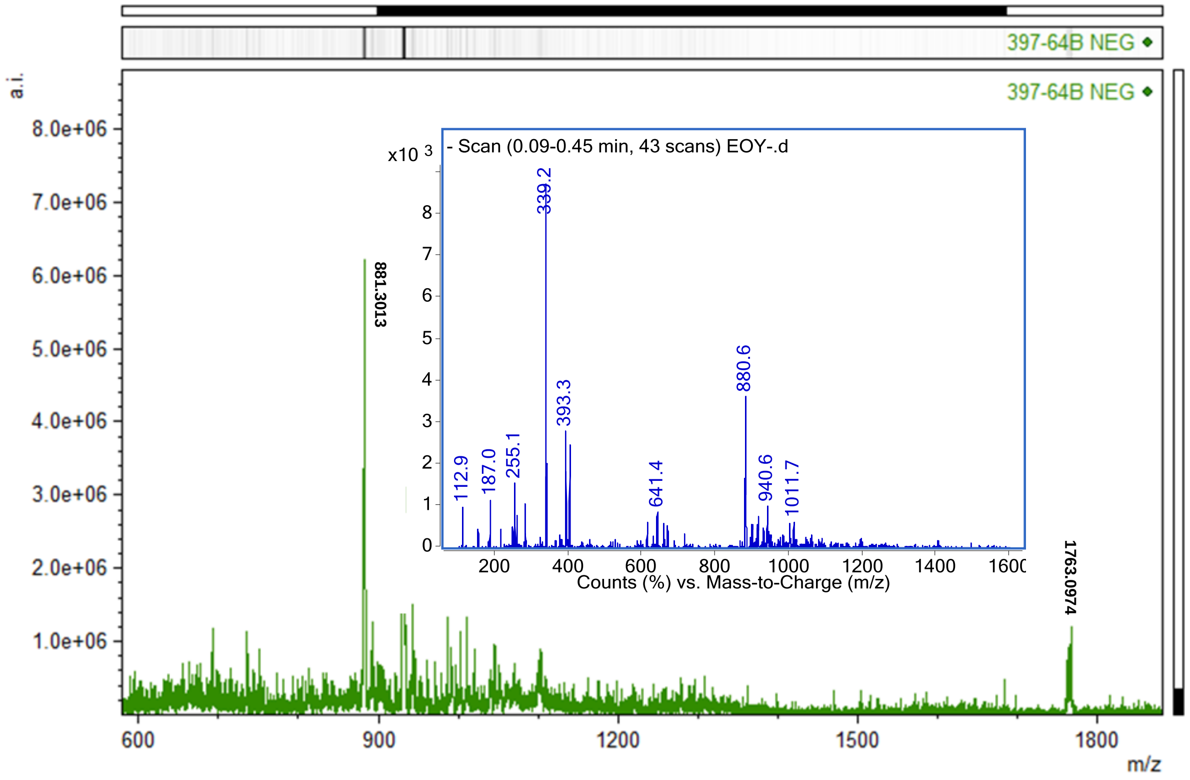

Figure S38: MS spectrum of EoY–β-CD (**2**–β-CD) conjugate in negative mode.

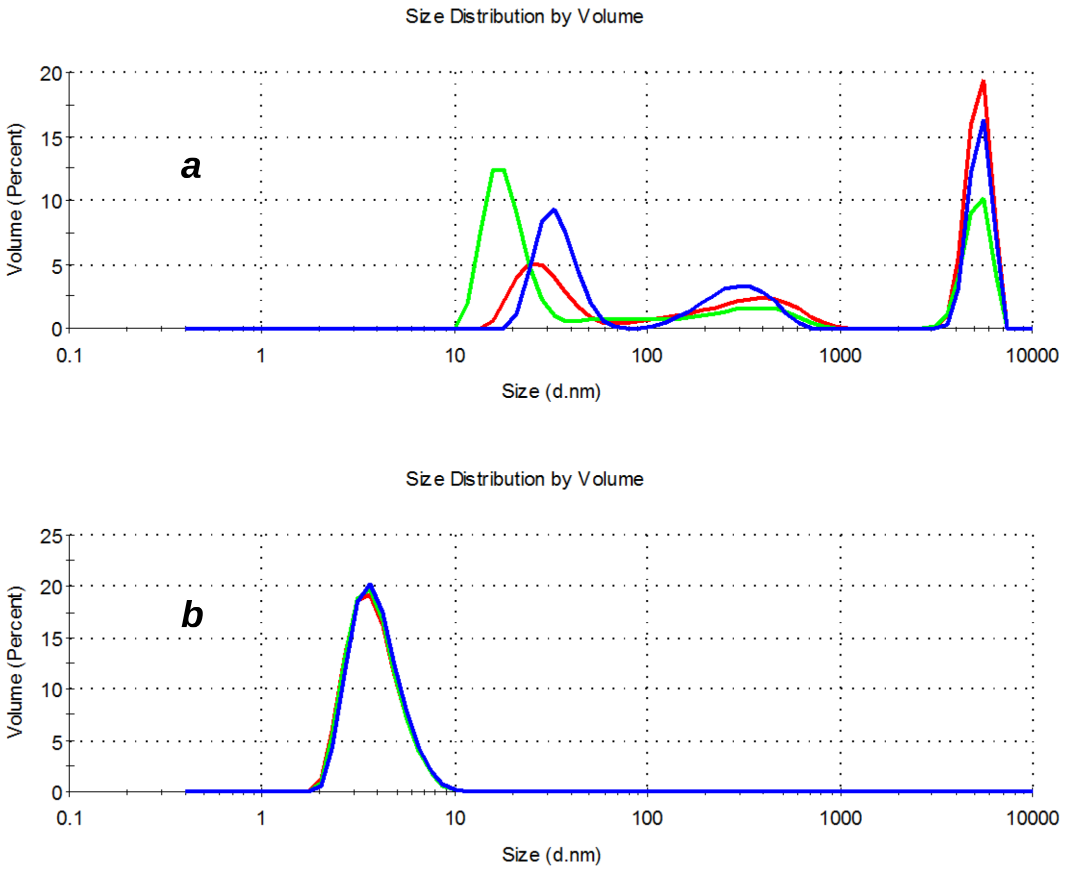

Figure S39: Size distributions of 1 mM aqueous (**a**) EoB–β-CD (**4**–β-CD) and (**b**) EoY–β-CD (**2**–β-CD) solutions at 25.0 °C (pH 7) by volume (three parallel measurements: blue, green and red lines).

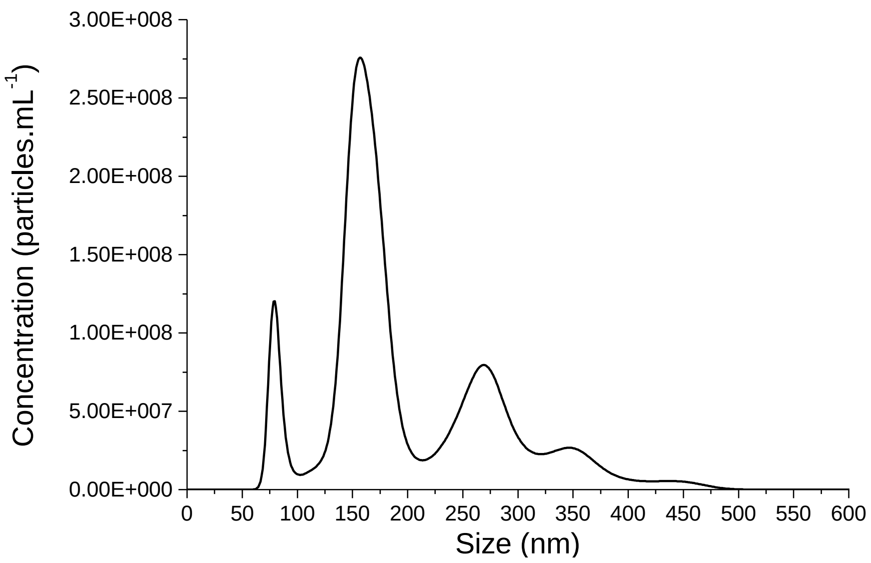

Figure S40: Size distributions of 1 mM aqueous EoB–β-CD (**4**–β-CD) solution at 25.0 °C (pH 7) by nanoparticles tracking analysis.

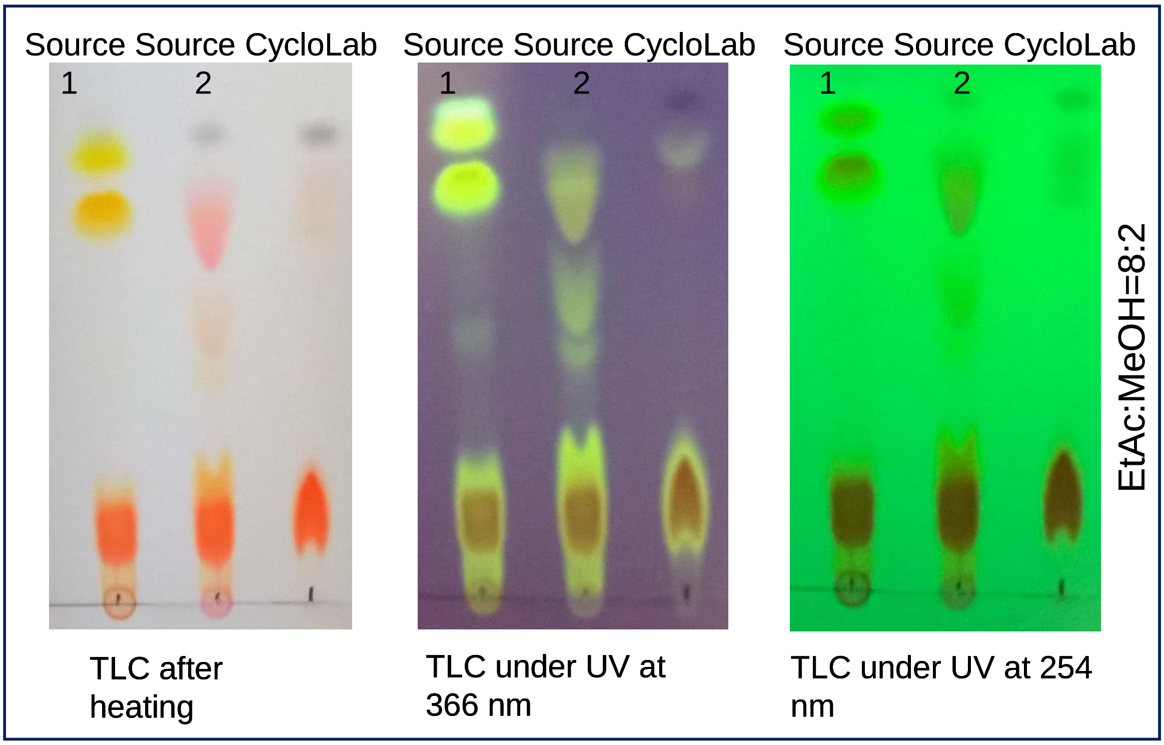

Figure S41: TLC evaluation of the purity of commercially available eosin Y from two different providers (source 1, source 2) and of EoY (**2**) synthesized in CycloLab.

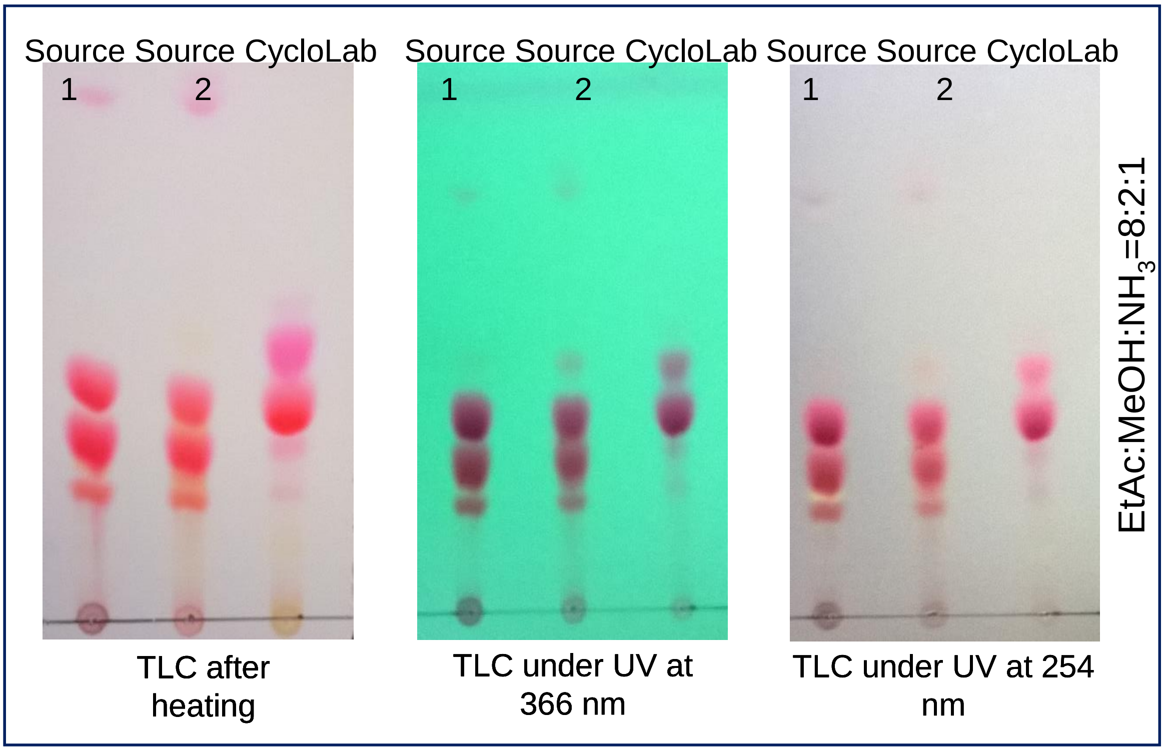

Figure S42: TLC evaluation of the purity of commercially available eosin B from two different providers (source 1, source 2) with EoB (**4**) synthesized in CycloLab.

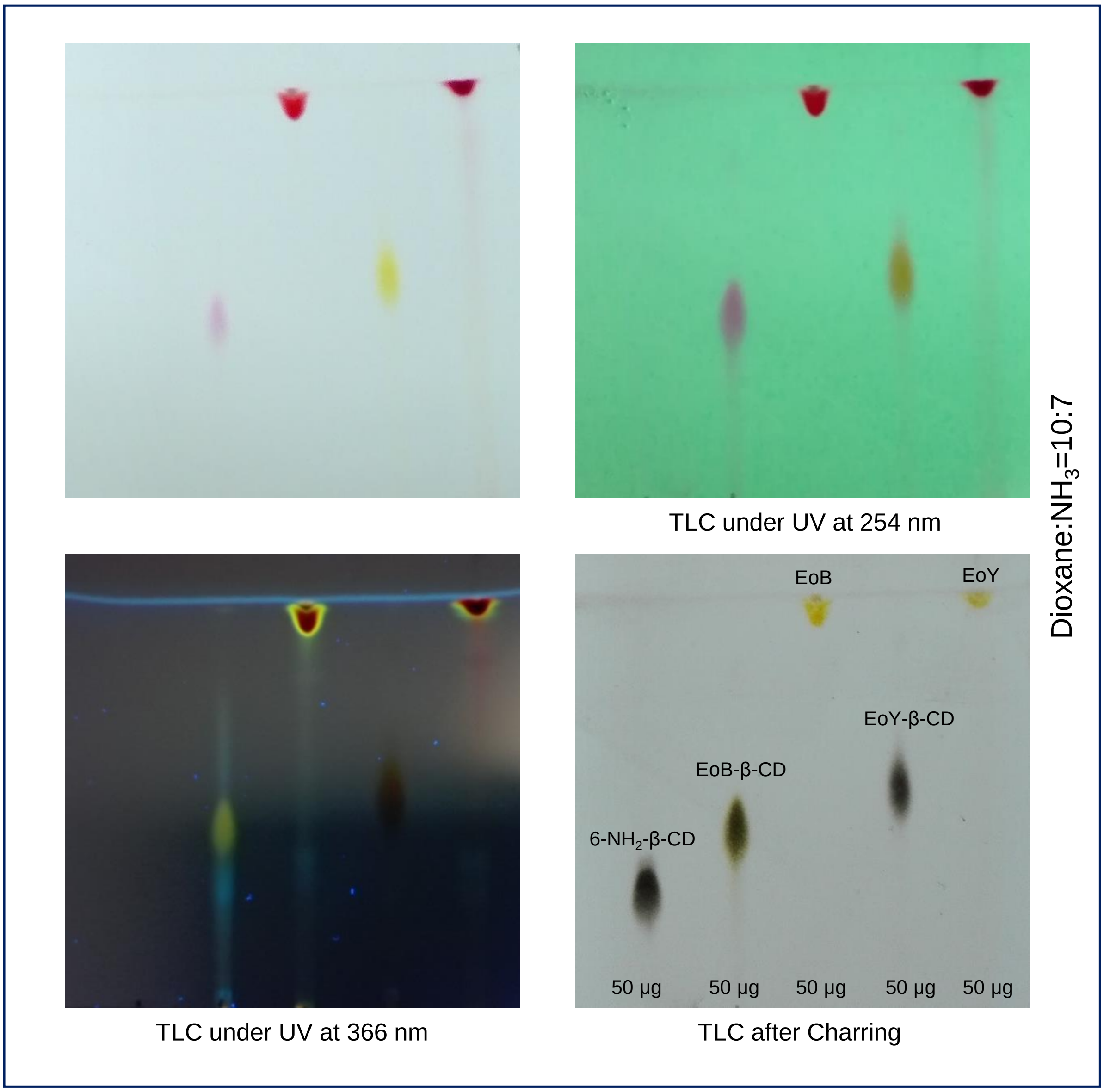

Figure S43: TLC evaluation of the purity of eosin-appended β-CDs with respect to 6-monoamino-β-CD, CD-related impurities and free dyes (EoB, **4** and EoY, **2**). For each compound, 5 µL of a 1% solution (w/V) (diluted wit 50% DMSO/H_2O) were spotted on the TLC plate; this volume corresponds approximatively to 50 µg of material.

In Figure S43 the results of the chromatographic purification are shown. The process allowed the exhaustive removal of the unreacted 6-monoamino-β-CD and the other CD-related impurities (see Figure 1 for comparison). Free dyes EoB (**4**) and EoY (**2**) are barely detectable in the final products under the examined conditions.
